# Respiratory syncytial virus seasonality and prevention strategy planning for passive immunisation of infants in low-income and middle-income countries: a modelling study

**DOI:** 10.1016/S1473-3099(20)30703-9

**Published:** 2021-09

**Authors:** You Li, David Hodgson, Xin Wang, Katherine E Atkins, Daniel R Feikin, Harish Nair

**Affiliations:** a Centre for Global Health, Usher Institute, University of Edinburgh, Edinburgh, UK; b Centre for Mathematics, Physics and Engineering in the Life Sciences and Experimental Biology, University College London, London, UK; c Faculty of Epidemiology and Population Health, London School of Hygiene & Tropical Medicine, London, UK; d Department of Immunizations, Vaccines, and Biologicals, WHO, Geneva, Switzerland; e Respiratory Syncytial Virus Network (ReSViNET) Foundation, Zeist, Netherlands

## Abstract

**Background:**

Respiratory syncytial virus (RSV) represents a substantial burden of disease in young infants in low-income and middle-income countries (LMICs). Because RSV passive immunisations, including maternal vaccination and monoclonal antibodies, can only grant a temporary period of protection, their effectiveness and efficiency will be determined by the timing of the immunisation relative to the underlying RSV seasonality. We aimed to assess the potential effect of different approaches for passive RSV immunisation of infants in LMICs.

**Methods:**

We included 52 LMICs in this study on the basis of the availability of RSV seasonality data and developed a mathematical model to compare the effect of different RSV passive immunisation approaches (seasonal approaches *vs* a year-round approach). For each candidate approach, we calculated the expected annual proportion of RSV incidence among infants younger than 6 months averted (effectiveness) and the ratio of per-dose cases averted between that approach and the year-round approach (relative efficiency).

**Findings:**

39 (75%) of 52 LMICs included in the study had clear RSV seasonality, defined as having more than 75% of annual RSV cases occurring in 5 or fewer months. In these countries with clear RSV seasonality, the seasonal approach in which monoclonal antibody administration began 3 months before RSV season onset was only a median of 16% (IQR 13–18) less effective in averting RSV-associated acute lower respiratory infection (ALRI) hospital admissions than a year-round approach, but was a median of 70% (50–97) more efficient in reducing RSV-associated hospital admissions per dose. The seasonal approach that delivered maternal vaccination 1 month before the season onset was a median of 27% (25–33) less effective in averting hospital admissions associated with RSV-ALRI than a year-round approach, but was a median of 126% (87–177) more efficient at averting these hospital admissions per dose.

**Interpretation:**

In LMICs with clear RSV seasonality, seasonal approaches to monoclonal antibody and maternal vaccine administration might optimise disease prevention by dose given compared with year-round administration. More data are needed to clarify if seasonal administration of RSV monoclonal antibodies or maternal immunisation is programmatically suitable and cost effective in LMICs.

**Funding:**

The Bill & Melinda Gates Foundation, World Health Organization.

## Introduction

Respiratory syncytial virus (RSV) represents a substantial burden of disease in young children (aged <5 years), particularly in low-income and middle-income countries (LMICs) and in infants younger than 6 months.^1^, ^2^ RSV activity is seasonal in most parts of the world and thus puts substantial pressure on health-care services during the seasonal epidemics.^3^ RSV activity shows a latitudinal gradient in the seasonal onset in each hemisphere; for example, in the northern hemisphere, RSV season usually starts in the late summer months in the tropics and starts in late autumn or early winter months in the temperate areas.^3^, ^4^

Currently, several RSV vaccine candidates and monoclonal antibodies are in late clinical development.^5^ Maternal RSV immunisation grants protection to infants passively by boosting naturally occurring, maternally derived antibodies. New, long-acting monoclonal antibodies grant protection to infants by directly injecting antibodies engineered to have extended half-lives (approximately 5 months).^6^ In 2020, a cost-effectiveness study based on hypothetical efficacy data suggested that both RSV long-acting monoclonal antibodies and maternal vaccination can potentially be optimal candidates for Gavi-eligible countries, depending on a country's willingness-to-pay values.^7^

**Research in context**

**Evidence before this study**

Respiratory syncytial virus (RSV) represents a substantial burden of disease in infants younger than 6 months in low-income and middle-income countries (LMICs). Several novel RSV prophylactic products are being developed to reduce RSV infections among young infants, including maternal vaccines and immunoprophylaxis. Because these products only provide protection for several months, RSV seasonality needs to be considered when implementing immunisation programmes to optimise their use. We searched PubMed with no language restrictions for any studies published before May 12, 2020, that assessed the role of RSV seasonality in the effectiveness and efficiency of novel RSV prophylactic programmes for infants in LMICs using the following search formula: (“respiratory syncytial virus” OR RSV) AND (impact OR effective* OR efficien* OR cost-effective*) AND (vaccine OR prophyla* OR antibod* OR immunisation OR immunization). We did not identify any studies that assessed the role of RSV seasonality in the RSV immunisation programmes for LMICs.

**Added value of this study**

To our knowledge, this is the first study to assess the effect of seasonal versus year-round passive immunisation strategies against RSV for infants in LMICs. In the LMICs with clear RSV seasonality, seasonal approaches prevented nearly as many RSV-associated hospital admissions (effectiveness) and more RSV-associated hospital admissions per dose (relative efficiency) compared with year-round administration. Results from a multiyear analysis suggested that the effectiveness and relative efficiency of these seasonal approaches could remain stable from year to year if countries applied the same seasonal administration schedules.

**Implications of all the available evidence**

Our results suggest that seasonal RSV prevention approaches might be considered in some LMICs with clear seasonality, but more information is needed on the cost-effectiveness and programmatic feasibility of seasonal administration.

Nonetheless, it should be noted that both maternal immunisation and monoclonal antibodies will only protect an infant for a limited period (approximately 3–5 months).^6^, ^8^ Therefore, seasonal dosing administration approaches in places with clear seasonality might enhance cost-effectiveness of these prophylaxis strategies. In this study, we assessed the potential effect of different approaches for administration of monoclonal antibodies and maternal vaccination among LMICs by evaluating the annual and per-dose proportion of RSV-associated acute lower respiratory infections (ALRIs) averted among infants younger than 6 months.

## Methods

### Data sources

We listed countries as LMICs (appendix pp 1–2) based on World Bank income classification (updated in June, 2019).^9^ 52 LMICs with available RSV seasonality data were included in our study. Data on RSV seasonality, burden, immunisation coverage, and immunisation efficacy were identified and extracted (details in appendix pp 3–6).

Briefly, we included RSV seasonality data from our recent systematic review^3^ and updated the literature search to include studies published between 2018 and 2019 (details in appendix pp 3–5). Because our study focuses on the national level effect of RSV prophylaxis, nationwide RSV activity data, where available, were selected to represent the RSV activity for a given country. If no nationwide RSV data were available, the nearest site with RSV activity data to the country's geographical centre was selected to represent its nationwide RSV activity. For RSV burden data, RSV-ALRI incidence and associated hospital admission rates among infants in LMICs were obtained from our previously published RSV global burden estimates.^1^

For the immunisation coverage data, we assumed that monoclonal antibodies were administered at birth, as this was considered a practical option for LMICs, and at the same country-specific coverage as other birth doses (BCG and hepatitis B vaccines).^10^ We assumed that maternal vaccine was administered at the beginning of the third trimester. We used the WHO ANC4+ indicator (defined as the proportion of women aged 15–49 years with a livebirth who received antenatal care four or more times)^11^ as a proxy for maternal vaccine coverage. We used available efficacy data for the candidate product furthest along in clinical trials—ResVax (an aluminium adjuvanted RSV fusion protein recombinant nanoparticle vaccine; Novavax)^8^ for maternal immunisation and nirsevimab (AstraZeneca/Sanofi)^6^ as the long-acting monoclonal antibody.

### Definition of RSV seasonal epidemics

We used the same definition for RSV seasonal epidemics as detailed in our 2019 study on global RSV seasonality.^3^ Briefly, annual average percentage, defined as the proportion of the estimated annual RSV incidence that occurred in each month, was calculated to describe the seasonality of RSV for each country. The duration of RSV seasonal epidemics was defined by the minimum number of months that accounted for at least 75% of the annual number of positive RSV cases, with each of these months labelled as an epidemic month. An inverse relationship existed between the duration of RSV seasonal epidemics and the degree of RSV seasonality, with shorter duration of RSV seasonal epidemics indicating greater seasonal activity. We defined countries with clear RSV seasonality as those that had 5 or fewer epidemic months in a year. For multiyear RSV activity data, the aforementioned definitions were applied to each year. For countries with clear RSV seasonality, the onset of the RSV season was defined by the first epidemic month of the longest consecutive epidemic months.

### Candidate approaches for RSV prophylaxis

**Figure 1:**
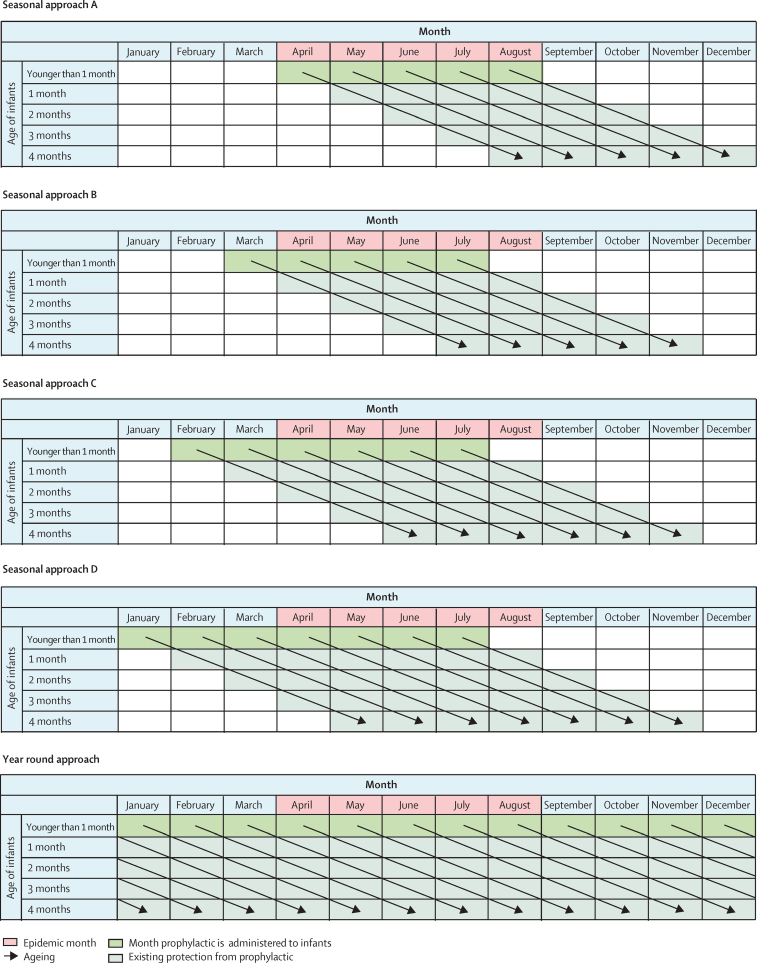
The candidate approaches for monoclonal antibody administration

For the monoclonal antibody programme, five candidate approaches were considered, including four seasonal approaches (A–D), and one year-round approach (figure 1). In seasonal approach A, monoclonal antibodies are administered in each epidemic month. Seasonal approaches B, C, and D begin administration of monoclonal antibodies before the onset of the first epidemic month (approach B, 1 month before onset; approach C, 2 months before onset; approach D, 3 months before onset), to protect infants who are born several months before the RSV season but are likely to be exposed to the virus during the RSV season at a very young age when the risk of severe RSV disease is high.

**Figure 2:**
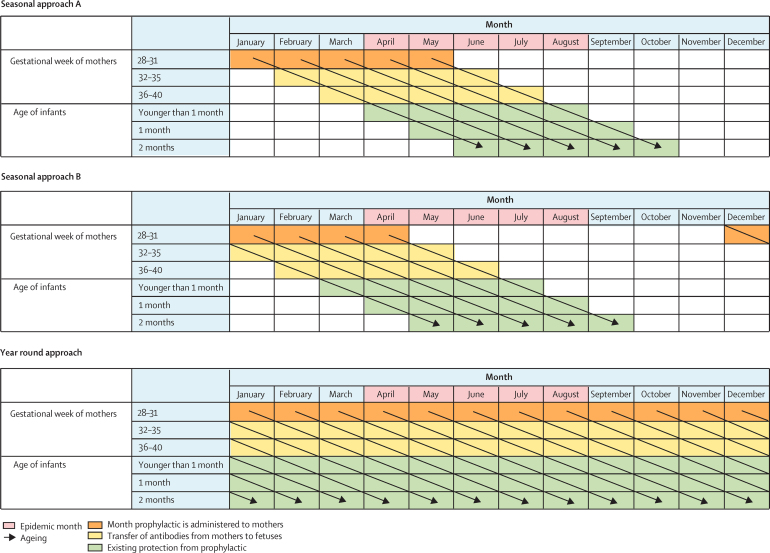
The candidate approaches for maternal vaccine administration

For the maternal vaccine programme, three candidate approaches were considered, including two seasonal approaches (A and B), and one year-round approach, the same as the corresponding approaches for the monoclonal antibody programme (figure 2). Unlike the monoclonal antibody programme, the maternal vaccine administration is timed according to the maternal due date, and we did not consider approach C or D for the maternal vaccine due to the likely shorter duration of protection by the maternal vaccine (approximately 90 days) than the monoclonal antibody (approximately 150 days). More details regarding the seasonal approaches are in the appendix (p 7). Note that for each seasonal approach, the number of dosing months (ie, months in which the administration of RSV prophylaxis needs to be implemented) is country-specific as it depends on the number of epidemic months.

### Definitions for effectiveness and relative efficiency

For each candidate approach, we defined effectiveness as the proportion of the total annual RSV-ALRIs and associated hospital admissions among infants younger than 6 months that could be averted. We defined relative efficiency as the ratio of per-dose effectiveness between the seasonal approach and the year-round approach; countries with more seasonal RSV activity were expected to have higher relative efficiency because fewer doses are required to prevent the same proportion of incidence and hospital admissions.

Detailed calculations related to effectiveness and relative efficiency are shown in the appendix (p 8). All calculations were done for each country, each RSV prophylaxis (ie, monoclonal antibody and maternal vaccine), and each RSV outcome (ie, RSV-ALRIs and associated hospital admissions). Briefly, we applied the monthly RSV activity to the annual RSV incidence in each month of age, assuming that RSV seasonality was identical across different months of age. Then, for each calendar month, the proportion of RSV episodes was calculated for each month of age (among infants younger than 6 months) so all the proportions across calendar months and months of age add up to 100%. Based on the dosing schedules, we identified the group of infants of specific months of age in specific months of a year who could directly benefit from the candidate programme, referred to as the benefit group (shown as existing protection from prophylactic in Figure 1, Figure 2, and month prophylactic is administered to infants in figure 1). Finally, we applied the corresponding efficacy and coverage results to the cumulative incidence among the benefit group to calculate the incidence that could be averted. For each approach, IQR was calculated to present the dispersion of effectiveness and relative efficiency among LMICs, with each LMIC as the unit.

In addition, we estimated the proportion of RSV-ALRI outcomes in the first 3 months of life by each birth month (appendix p 9).

### Sensitivity analysis and software

For a subset of LMICs for which multiyear RSV activity data were available, we calculated effectiveness and relative efficiency for each year using the same dosing schedule to assess the robustness of our results. As a sensitivity analysis, we considered 100% coverage to estimate an upper limit of the potential effectiveness. As protection from both monoclonal antibody and maternal vaccine might decay exponentially after birth, we also considered a monthly efficacy decay rate of 0·8 while maintaining the average efficacy consistent with the clinical trial efficacy data. For the maternal vaccine, we ran additional analyses by increasing the duration of protection from 3 months to 5 months and by assuming the same efficacy as the monoclonal antibody (details in appendix p 10).

All data analyses were done using R software (version 3.5.2) with codes available in GitHub.^12^ Workflows are described in the appendix (p 25).

### Role of the funding source

The funder of the study was involved in study design, data interpretation, and writing of the manuscript. The funder of the study had no role in data collection or data analysis.

## Results

52 LMICs were included in the analysis (appendix p 25). Most of these LMICs (39 [75%]) had clear seasonal RSV activity. Equatorial LMICs tended to have year-round RSV activity. Countries with similar latitudes were more likely to have similar RSV seasonality (appendix p 26). Multiyear RSV activity data were available in 25 LMICs. In countries with clear RSV seasonality, RSV onset was within 1 month (before or after) of the country's average onset in 113 (74%) of the 152 years and was within 2 months (before or after) of the country's average onset in 135 (89%) of the years (appendix p 27).

Infants born 1–2 months before peak RSV activity had the highest risk of being admitted to hospital due to RSV-ALRI, as shown by a comparison of the proportion of children born in each month and the peak month of RSV-ALRI hospitalisation (appendix p 28). Similar time lags were observed between birth month and RSV-ALRI incidence (appendix p 29).

The median number of monoclonal antibody dosing months among the 52 LMICs was 4 months (IQR 3–5) for seasonal approaches A and B, 6 months (4–7) for seasonal approach C, and 6 months (5–8) for seasonal approach D (appendix p 30). The median number of maternal vaccine dosing months was 4 months (3–6) for seasonal approaches A and B (appendix p 31).

**Figure 3:**
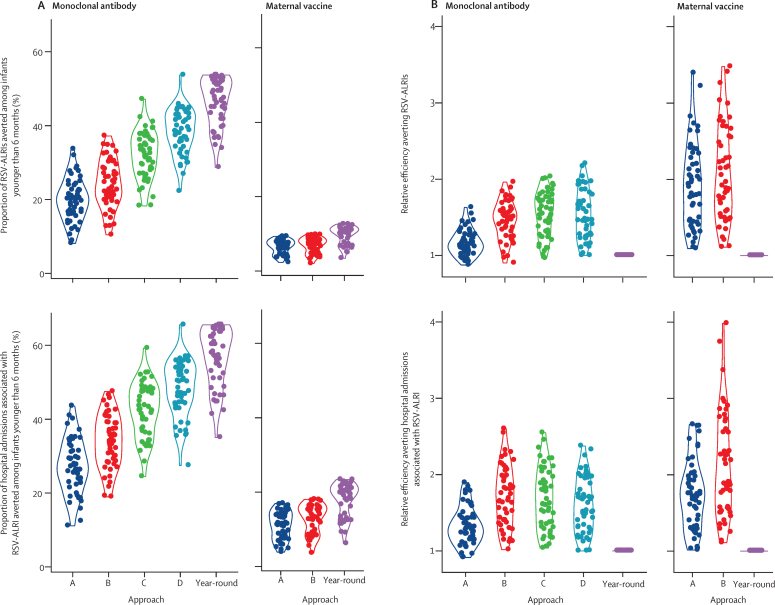
Effectiveness and relative efficiency for monoclonal antibodies and maternal immunisation in LMICs (A) Effectiveness for monoclonal antibodies and maternal vaccination. (B) Relative efficiency for monoclonal antibodies and maternal vaccination. Each point represents one country, the width of the curve corresponds with the approxiamte frequency of data points. Effectiveness is defined by annual proportion averted among infants younger than 6 months; relative efficiency is defined by the ratio between per-dose effectiveness of a seasonal approach and that of the year-round approach. LMICs=low-income and middle-income countries. RSV-ALRI=respiratory syncytial virus-associated acute lower respiratory infection.

We evaluated effectiveness and relative efficiency across all 52 countries (figure 3). For the monoclonal antibody, the effectiveness against both RSV-ALRI incidence and associated hospital admissions was highest in the year-round approach and seasonal approach D, followed by seasonal approach C, and was lowest in seasonal approaches A and B. Relative efficiency for both RSV outcomes was highest in seasonal approaches B, C, and D, followed by seasonal approach A, and was lowest in the year-round approach. For the monoclonal antibody, the median effectiveness of the year-round approach for averting hospital admissions associated with RSV-ALRI was 58·1% (IQR 51·3–63·8), and increased to 66·2% (66·2–66·2) when assuming 100% coverage (appendix p 11).

For the maternal vaccination, seasonal approach B had the highest relative efficiency. The median effectiveness of the year-round approach for averting hospital admissions associated with RSV-ALRI was 19·4% (IQR 13·1–21·1; appendix p 11), increasing to 23·6% (23·6–23·6) when assuming 100% coverage. In sensitivity analyses, median year-round effectiveness increased slightly to 20·7% (18·3–22·8) when assuming 5 months of protection and to 34·2% (23·2–37·3) when assuming the same efficacy as the monoclonal antibody.

**Figure 4:**
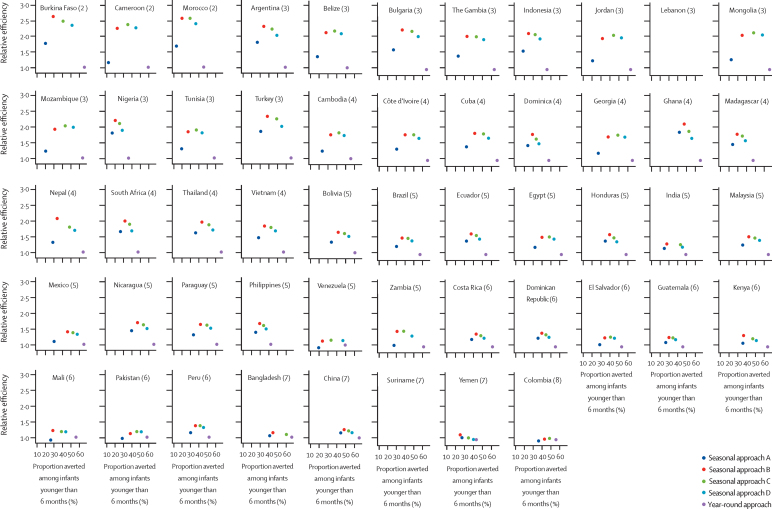
Country-specific effectiveness and relative efficiency of monoclonal antibodies in averting hospital admission associated with RSV-ALRIs The number in parentheses after each country indicates the duration of RSV epidemics (in months). Effectiveness is defined by annual proportion averted among infants younger than 6 months; relative efficiency is defined by the ratio between per-dose effectiveness of a seasonal approach and that of the year-round approach. Approaches in the upper right quadrant of each chart would be considered those with optimal effectiveness and relative efficiency. RSV=respiratory syncytial virus. RSV-ALRI=respiratory syncytial virus-associated acute lower respiratory infection.

Results of the assessment of the effectiveness and relative efficiency by country are shown in figure 4 and appendix p 32 for the monoclonal antibody, and in appendix pp 33–34 for the maternal vaccine. For the monoclonal antibody, in countries with an RSV epidemic with a duration of 5 or fewer months, seasonal approach D was generally favourable, with a median relative efficiency of 1·70 (IQR 1·50–1·97) in preventing hospital admissions associated with RSV-ALRI and a median loss of effectiveness of only 16% (IQR 13–18) compared with the year-round approach (table). As the duration of RSV epidemics increased, the advantages of seasonal approaches became less pronounced and the year-round approach became more favourable (figure 4; appendix p 32). Similar findings were observed for maternal immunisation (appendix pp 33–34). These results were robust when assuming a monthly efficacy decay rate of 0·8 (appendix p 12).

**Table:** Effectiveness and relative efficiency for each candidate approach among countries with 5 or fewer epidemic months

	**Number of dose months**	**RSV-ALRI incidence**			**Hospital admission associated with RSV-ALRI**		
		Effectiveness	Effectiveness ratio*	Relative efficiency	Effectiveness	Effectiveness ratio*	Relative efficiency
**Monoclonal antibody**							
Seasonal approach A	4 (3–5)	18·1% (14·5–21·9)	0·39 (0·32–0·46)	1·19 (1·09–1·30)	25·8% (20·3–30·7)	0·45 (0·37–0·53)	1·36 (1·24–1·54)
Seasonal approach B	4 (3–5)	22·3% (19·3–28·0)	0·48 (0·40–0·59)	1·55 (1·46–1·70)	33·0% (28·2–39·0)	0·58 (0·51–0·65)	1·83 (1·65–2·10)
Seasonal approach C	5 (4–6)	32·1% (26·9–36·2)	0·68 (0·60–0·73)	1·69 (1·52–1·80)	42·4% (36·4–48·0)	0·75 (0·70–0·78)	1·82 (1·60–2·09)
Seasonal approach D	6 (5–7)	38·6% (34·7–42·9)	0·82 (0·79–0·84)	1·65 (1·46–1·89)	49·4% (44·3–53·8)	0·84 (0·82–0·87)	1·70 (1·50–1·97)
Year-round	12 (12–12)	49·1% (42·2–52·5)	1 (ref)	1 (ref)	59·9% (51·5–64·1)	1 (ref)	1 (ref)
**Maternal vaccine**							
Seasonal approach A	4 (3–5)	6·8% (5·2–7·7)	0·66 (0·59–0·70)	2·03 (1·79–2·40)	11·1% (7·8–13·7)	0·60 (0·51–0·67)	1·82 (1·69–2·07)
Seasonal approach B	4 (3–5)	7·1% (5·3–8·2)	0·70 (0·64–0·75)	2·22 (1·85–2·69)	13·7% (10·6–15·8)	0·73 (0·67–0·75)	2·26 (1·87–2·77)
Year-round	12 (12–12)	10·4% (8·8–11·2)	1 (ref)	1 (ref)	19·6% (16·4–21·0)	1 (ref)	1 (ref)

Data are median (IQR). RSV-ALRI=respiratory syncytial virus-associated acute lower respiratory infection.

* Effectiveness ratio is the ratio between the effectiveness of the seasonal approach and that of the year-round approach.

We assessed the year-to-year variations in the effectiveness and relative efficiency of RSV immunisations, assuming countries adopted fixed dosing schedules on the basis of their average epidemic months. In countries with clear RSV seasonality, seasonal approaches C and D for the monoclonal antibody and seasonal approach B for the maternal vaccine had stable efficiency and effectiveness results (appendix pp 13–24, 35–38).

## Discussion

To the best of our knowledge, this is the first study to compare the effect of seasonal programmes and year-round programmes for RSV prophylactic products targeted at infants younger than 6 months in LMICs with variable RSV seasonality. RSV activity was clearly seasonal (75% of disease episodes occur over ≤5 months) in three-quarters of the LMICs. In these LMICs with clear RSV seasonality, seasonal immunisation approaches achieved high relative efficiency and did not lose substantial effectiveness compared with the year-round approach for both monoclonal antibody and maternal vaccine strategies. Moreover, these results are insensitive to annual variation in RSV seasonality within countries.

Previous studies on global RSV seasonality^3^, ^4^ have shown that in countries with 5 or fewer RSV epidemic months, a single dose of long-acting passive immunisation or maternal immunisation could protect infants for most of the duration of the peak RSV season. This was supported by the effectiveness and relative efficiency results in the present study among countries with clear RSV seasonality. With a median programme length of 6 months (IQR 5–8), the monoclonal antibody approach in which vaccination began 3 months before the RSV season could prevent a median of 49·4% (44·3–53·8) of the hospital admissions associated with RSV-ALRI among infants younger than 6 months in these countries. Compared with the year-round approach, this was a median of 70% (50–97) more efficient by cutting down approximately half of the doses demanded and averting a median of only 16% (13–18) fewer admissions to hospital than the year-round approach. For the maternal vaccine, with a median programme length of 4 months (3–5) per year, the optimal seasonal approach could prevent 13·7% (10·6–15·8) of the hospital admissions associated with RSV-ALRI among infants younger than 6 months in countries with clear RSV seasonality. Compared with the year-round approach, this approach was a median of 126% (87–177) more efficient by cutting down almost two-thirds of the doses demanded and averting only 27% (25–33) fewer admissions to hospital associated with RSV-ALRI than the year-round approach.

Among all seasonal approaches in the study, the approaches that advance immunisation by 1–3 months before the onset of RSV seasonal epidemics were generally more effective and efficient than the approach that only administered a prophylactic during RSV epidemic months. This finding was similar to the mathematical modelling study in the UK by Cromer and colleagues.^13^ Instead of proposing seasonal strategies, Cromer and colleagues applied a grid search that went through all combinations of dosing schedules from a 1-month programme to a year-round programme and from January to December. They found that the most cost-effective strategy was to protect neonates born in November, 1 month before the RSV season. In the present study, we found that infants born 1–2 months before RSV seasonal epidemics in LMICs generally had a higher risk of being admitted to hospital for RSV-ALRI during the first 3 months of their life (appendix p 28). This finding supports the advanced seasonal approaches for immunisation programmes that aim to protect neonates.

Instead of using hypothetical vaccine efficacy data, we applied real-world efficacy data from clinical trials. As no efficacy data were available in terms of the change over time during the period of protection, we assumed the efficacy remained at the same level over time in our main analysis. This assumption might not hold as efficacy is likely to decay over the first few months of life as the antibody level decreases, which would disproportionally affect advanced seasonal approaches because the protection might decay to a lower level when the infants entered the RSV season 1–3 months after birth. Nonetheless, our results from sensitivity analyses using a decay rate of 0·8 showed little sign of effect on the effectiveness and efficiency results of these advanced seasonal approaches. Another potential limitation of the efficacy data used for this analysis is that they were derived from clinical trials that enrolled participants mostly in high-income and upper-middle-income countries (albeit in resource-poor settings in upper-middle-income countries); efficacy data from populations in low-income countries could improve the validity of this analysis.

Although LMICs have a higher burden of RSV-ALRI than high-income countries, most have no ongoing RSV surveillance to inform decision making on their RSV immunisation strategy. In this study, we included multiyear RSV data from 25 LMICs and found that for countries with clear RSV seasonality, the onset of RSV season varied by 1 month in most (113 [74%] of 152) of the years. Similar findings were observed in the early report of the WHO RSV surveillance pilot.^14^ The relative stability of RSV season from year to year in LMICs with clear RSV seasonality suggest that a few years of surveillance to establish seasonality might be sufficient to establish a fixed seasonal administration programme.

Although the use of seasonal approaches for countries with clear RSV seasonality was supported by both monoclonal antibody and maternal vaccine immunisations, the maternal vaccine had relatively low effectiveness in the results of our study. Results from our prespecified sensitivity analyses suggest that the efficacy of the maternal vaccine has a determinant role in the effectiveness of a maternal vaccination programme, which increased by 76·3% (from 19·4% [IQR 13·1–21·1] to 34·2% [23·2–37·3]) when applying a higher efficacy (equivalent to the monoclonal antibody). Of note, the vaccine efficacy used for the maternal immunisation approach was based on ResVax, which was the first RSV vaccine to show efficacy in a phase 3 trial (prevention of hospitalisation; a secondary outcome), but which did not meet the primary endpoint of the same trial, which was to prevent medically significant lower respiratory infection.^8^ Future trials of maternal immunisation products might result in higher efficacy inputs for this approach. It should also be noted that the efficacy results were assumed not to differ by calendar month; as a result, when calculating relative efficiency, the efficacy terms in seasonal approaches and the year-round approach were cancelled out in the formula (appendix pp 8–9).

There are some caveats when interpreting the results of this study. First, the coverage data we applied to the analysis might not reflect the real uptake and thus might bias the estimate for effectiveness. For the monoclonal antibody, although the birth dose of BCG vaccine and hepatitis B vaccine are both likely to be valid proxies, concerns among parents about their newborn babies having another injection could lead to a lower uptake. For the maternal vaccine, ANC4+ data are likely to underestimate the uptake because pregnant women with four or fewer antenatal care visits could still present for an earlier visit during the eligible vaccination window. Nonetheless, sensitivity analyses that assumed 100% coverage and that assumed the same efficacy as the monoclonal antibody suggested that effectiveness was less sensitive to coverage than to other factors.

Second, we were unable to adjust for different timings of maternal immunisation due to the absence of relevant efficacy data. Earlier immunisation increased maternal vaccine-induced antibodies and was expected to be associated with higher efficacy.^15^ We assumed that all maternal vaccines were administered in the 28th gestational week, which could lead to a possible underestimate for the effectiveness. This is because the vaccine efficacy data were extracted from the ResVax trial that included mothers who were vaccinated after the 33rd gestational week (thus having lower efficacy than if they were to be vaccinated in the 28th gestational week).^8^ Similarly, we were unable to adjust for the potential effect of preterm delivery on the effectiveness and relative efficiency of maternal immunisation. Preterm delivery leads to lower efficacy by reducing the time for the production of vaccine-induced antibodies and for the transfer of these antibodies to the fetus.^15^, ^16^ Additionally, for the seasonal approaches to maternal immunisation, some prematurely born infants might not receive the vaccine because their expected date of delivery put them outside of the window for maternal vaccination.

Third, our analysis was done at the national level. For geographically large countries or countries with known high spatial variations in RSV season (eg, Kenya^3^), region-specific seasonal programmes or national year-round programmes might be considered. For 22 countries (42% of the included countries) with no available nationwide RSV seasonality data, we used the nearest site with available RSV seasonality data to each country's geographical centre as the best available proxy for nationwide RSV activity, assuming that there was a latitudinal and longitudinal gradient in the timing of RSV season onset within each country. This assumption might not hold true and nationwide RSV seasonality data are still needed from these countries to confirm our findings.

Fourth, the RSV seasonality data included in the study were from various sources and their representativeness could vary depending on criteria for testing, respiratory samples, and testing and reporting practices. These data were all collected before the COVID-19 pandemic.^17^ It is unknown how COVID-19 will affect the seasonality of RSV in the short and medium term (next 3–5 years).

Given the disproportionally high severe RSV disease burden in infants younger than 6 months in LMICs, it is crucial to consider the introduction of RSV passive immunisations to LMICs as soon as they become available. Our study showed that seasonal dosing approaches in LMICs with clear RSV seasonality might prevent more cases per dose administered than year-round administration. Such approaches might be more cost effective and feasible in supply-constrained settings. However, more information on the programmatic suitability and acceptability of seasonal approaches is warranted.

## Data sharing

The respiratory syncytial virus seasonality data from our literature search update are made freely available at https://github.com/leoly2017/RSV_seasonality_LMIC. All other data included in the analysis are publicly available and properly cited.

## Declaration of interests

YL has received grants from WHO relating to the current study, and grants from the Foundation for Influenza Epidemiology outside the submitted work. DH and KEA have received grants from WHO relating to the current study. HN has received grants from WHO relating to the current study. HN has also received grants from the Foundation for Influenza Epidemiology, Innovative Medicines Initiative, WHO, and National Institute of Health Research; personal fees from The Bill & Melinda Gates Foundation, Janssen, and AbbVie; and grants and personal fees from Sanofi, all outside the submitted work. All other authors declare no competing interests. The authors alone are responsible for the views expressed in this article and they do not necessarily represent the views, decisions, or policies of the institutions with which they are affiliated.
