# Supplementary material for: Respiratory syncytial virus seasonality and prevention strategy planning for passive immunisation of infants in low-income and middle-income countries: a modelling study
Source: Lancet Infect Dis. 2021 Sep;21(9):1303–12. doi: 10.1016/S1473-3099(20)30703-9 (PMC8386346; doi:10.1016/S1473-3099(20)30703-9)
Supplement: Supplementary appendix [file mmc1.pdf]

THE LANCET
Infectious Diseases

Supplementary appendix

This appendix formed part of the original submission and has been peer reviewed. We post it as supplied by the authors.

Supplement to: Li Y, Hodgson D, Wang X, Atkins KE, Feikin DR, Nair H. Respiratory syncytial virus seasonality and prevention strategy planning for passive immunisation of infants in low-income and middle-income countries: a modelling study. *Lancet Infect Dis* 2021; published online May 6. https://doi.org/10.1016/S1473-3099(20)30703-9.

Contents

List of low and middle income countries (LMIC) ... 1
Low-income economies ... 1
Lower-middle economies ... 1
Upper-middle economies ... 2
Detailed description of data included in the analysis ... 3
RSV activity data ... 3
Inclusion criteria ... 3
Exclusion criteria ... 3
Results of updated literature review ... 3
RSV burden data ... 6
RSV prophylactic coverage data ... 6
Efficacy data ... 6
Details on the candidate seasonal approaches ... 7
mAb ... 7
Maternal vaccine ... 7
Mathematical modelling ... 8
Notation ... 8
Calculations related to the effectiveness and relative efficiency ... 8
Calculations related to RSV-ALRI hospitalisations by birth month ... 9
Summary of the base case values and the values for sensitivity analyses ... 10
Supplementary tables of results ... 11
Table S1. Effectiveness and relative efficiency for each mAb candidate approach ... 11
Table S2. Effectiveness and relative efficiency for each maternal vaccine candidate approach ... 11
Table S3. Effectiveness and relative efficiency for each mAb candidate approach among countries with ⩽5 epidemic months, with monthly efficacy decay rate of 0.8 ... 12
Table S4. Effectiveness and relative efficiency for each maternal vaccine candidate approach in countries with ⩽5 epidemic months, with monthly efficacy decay rate of 0.8 ... 12
Table S5. Year-to-year variations in relative effectiveness and relative efficiency for each monoclonal antibodies candidate approach in countries with ⩽5 epidemic months ... 13
Table S6. Year-to-year variations in the effectiveness and relative efficiency for each maternal vaccine candidate approach in countries with ⩽5 epidemic months ... 13
Table S7. Country-specific year-to-year variations in the effectiveness and relative efficiency for each monoclonal antibodies candidate approach ... 14
Table S8. Country-specific year-to-year variations in the effectiveness and relative efficiency for each maternal vaccine candidate approach ... 20

Table S9. Year-to-year variations in relative effectiveness and relative efficiency for each monoclonal antibodies candidate approach in countries with ⩽5 epidemic months, with a monthly efficacy decay rate of 0.8 24
Table S10. Year-to-year variations in relative effectiveness and relative efficiency for each maternal vaccine candidate approach in countries with ⩽5 epidemic months with a monthly efficacy decay rate of 0.8 24
Supplementary figures 25
Figure S1. Schematic figure showing the workflow of the study 25
Figure S2. RSV activity data availability in LMICs 25
Figure S3. Month-by-month activity of RSV in LMICs. 26
Figure S4. Month-by-month activity of RSV in LMICs. 27
Figure S5. Distribution of RSV-ALRI hospitalisation in infants <3m by birth month and by calendar month 28
Figure S6. Distribution of RSV-ALRI in infants <3m by birth month and by calendar month 29
Figure S7. Dosing schedules for seasonal mAb programmes 30
Figure S8. Dosing schedules for seasonal maternal vaccine programmes 31
Figure S9. Country-specific results of effectiveness and relative efficiency in averting RSV-ALRI for monoclonal antibodies 32
Figure S10. Country-specific results of effectiveness and relative efficiency in averting RSV-ALRI hospitalisation for maternal vaccine 33
Figure S11. Country-specific results of effectiveness and relative efficiency in averting RSV-ALRI for maternal vaccine 34
Figure S12. Year-to-year variations of effectiveness and relative efficiency in averting RSV-ALRI for monoclonal antibodies 35
Figure S13. Year-to-year variations of effectiveness and relative efficiency in averting RSV-ALRI for monoclonal antibodies 36
Figure S14. Year-to-year variations of effectiveness and relative efficiency in averting RSV-ALRI hospitalisation for maternal vaccine 37
Figure S15. Year-to-year variations of effectiveness and relative efficiency in averting RSV-ALRI for maternal vaccine 38
GATHER checklist 39
Reference 41

List of low and middle income countries (LMIC)

The list of low and middle income countries are extracted from the World Bank Classifications by Income.[1]

Low-income economies

Afghanistan	Guinea-Bissau	Sierra Leone
Benin	Haiti	Somalia
Burkina Faso	Korea, Dem. People's Rep.	South Sudan
Burundi	Liberia	Syrian Arab Republic
Central African Republic	Madagascar	Tajikistan
Chad	Malawi	Tanzania
Congo, Dem. Rep	Mali	Togo
Eritrea	Mozambique	Uganda
Ethiopia	Nepal	Yemen, Rep.
Gambia, The	Niger	
Guinea	Rwanda	

Lower-middle economies

Angola	India	Papua New Guinea
Bangladesh	Indonesia	Philippines
Bhutan	Kenya	São Tomé and Principe
Bolivia	Kiribati	Senegal
Cabo Verde	Kyrgyz Republic	Solomon Islands
Cambodia	Lao PDR	Sudan
Cameroon	Lesotho	Timor-Leste
Comoros	Mauritania	Tunisia
Congo, Rep.	Micronesia, Fed. Sts.	Ukraine
Côte d'Ivoire	Moldova	Uzbekistan
Djibouti	Mongolia	Vanuatu
Egypt, Arab Rep.	Morocco	Vietnam
El Salvador	Myanmar	West Bank and Gaza
Eswatini	Nicaragua	Zambia
Ghana	Nigeria	Zimbabwe
Honduras	Pakistan	

Upper-middle economies

Albania	Fiji	Namibia
Algeria	Gabon	Nauru
American Samoa	Georgia	North Macedonia
Argentina	Grenada	Paraguay
Armenia	Guatemala	Peru
Azerbaijan	Guyana	Romania
Belarus	Iran, Islamic Rep.	Russian Federation
Belize	Iraq	Samoa
Bosnia and Herzegovina	Jamaica	Serbia
Botswana	Jordan	Sri Lanka
Brazil	Kazakhstan	South Africa
Bulgaria	Kosovo	St. Lucia
China	Lebanon	St. Vincent and the Grenadines
Colombia	Libya	Suriname
Costa Rica	Malaysia	Thailand
Cuba	Maldives	Tonga
Dominica	Marshall Islands	Turkey
Dominican Republic	Mauritius	Turkmenistan
Equatorial Guinea	Mexico	Tuvalu
Ecuador	Montenegro	Venezuela, RB

Detailed description of data included in the analysis

RSV activity data

Available RSV monthly activity data in LMICs were obtained from three sources, including systematic literature review, online datasets and RSV Global Epidemiology Network (RSV GEN), as detailed previously.[2] We updated the literature search to the end of 2019 using the previous search strategy[2] to capture new RSV studies published between 1st January 2018 and 31st December 2019. The literature review update was conducted by YL and XW, independently. We revisited our previous RSV seasonality dataset[2] to identify any data records that had available RSV activity data for three or more consecutive years for the multi-year analysis of this study. Detailed selection criteria for RSV activity data are attached below.

Inclusion criteria

- Laboratory-confirmed cases
- Number of RSV positives cases aggregated at least on a monthly basis
- At least 25 RSV positive cases per year
- Data available for at least consecutive 12 months, or at least consecutive 36 months for multi-year analysis

Exclusion criteria

- RSV data from only patients with comorbidities
- RSV data from only hospital-acquired infections

Results of updated literature review

After removal of duplicates, a total of 3000 publications were screened by title and abstract, and subsequently 269 full-text articles were screened. A total of 16 studies[3-18] (including data from 11 countries of which 6 were new) had eligible RSV seasonality data for LMICs (PRISMA flowchart on Page 4). Details of these studies are presented below (Page 5).

The 16 new studies were assessed for data quality, and data were extracted as described previously.[2]

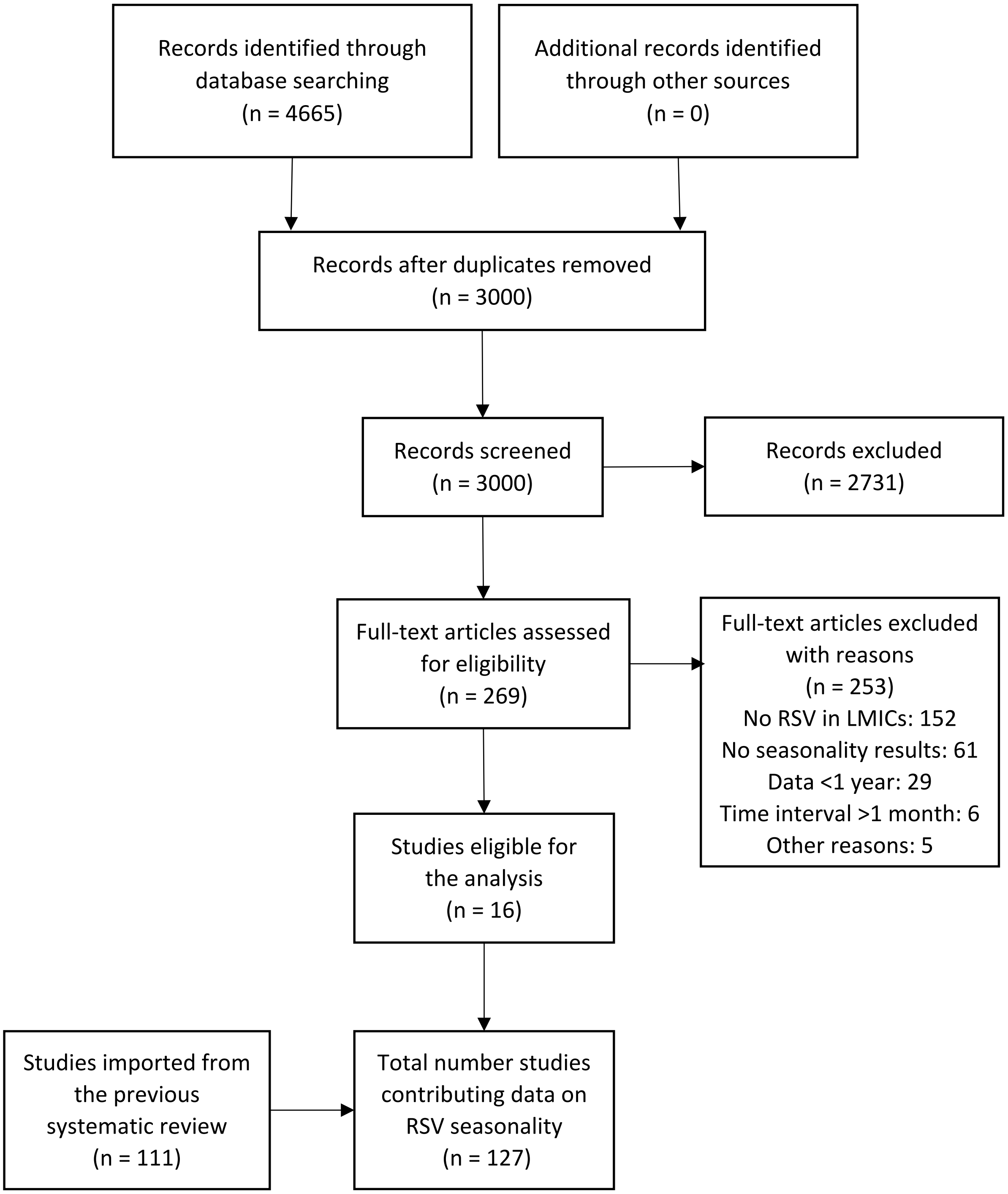

PRISMA flowchart showing the search and selection of studies for the updated literature review (contextualising the previous published systematic review[2]). Other reasons for exclusion include subset of online reports (2), no full-text (1), outbreak investigation data only (1) and review (1).

Summary of studies reporting RSV seasonality in LMICs from the literature update

Data provider	Site	Country	Longitude	Latitude	Case definition	Testing methods	Total RSV cases
Appak, 2019[3]	Izmir	Turkey	27.1	38.4	ARI	PCR	292
Chakhunashvili, 2018[4]	nationwide	Georgia	44.0	41.7	SARI	PCR	251
Chavez, 2019[5]	La Paz	Bolivia	–68.1	–16.5	SARI	PCR	116
Chittaganpitch, 2018[6]	nationwide	Thailand	100.8	14.9	ILI	PCR	699
Kadjo, 2018[7]	nationwide	Côte d'Ivoire	–5.5	7.5	ILI and SARI	PCR	75
Korsun, 2019[8]	nationwide	Bulgaria	25.5	42.6	ALRI	PCR	90
Li, 2018[9]	Gansu	China	104.3	35.8	ARI	PCR	122
Li, 2019[10]	Guangzhou	China	113.3	23.1	Severe ALRI	PCR	75
Liang, 2019[11]	Lanzhou	China	103.8	36.1	ARI	PCR	1272
Obodai, 2018[12]	Accra	Ghana	-0.2	5.6	ALRI	PCR	114
Oladele, 2019[13]	Ilorin	Nigeria	4.5	8.5	ALRI	Antigen Test	41
Rabarison, 2019[14]	Analamanga	Madagascar	47.5	–18.9	SARI	NA	413
Teck, 2019[15]	Kelantan	Malaysia	102.1	5.3	ALRI	Antigen test	93
Xu, 2018[16]	Tianjin	China	117.2	39.1	SARI	PCR	29
Yu, 2019[17]	Beijing	China	116.4	39.9	pneumonia	PCR	1270
Zeng, 2018[18]	Nanjing	China	118.8	32.1	respiratory diseases	serological	26

ARI = acute respiratory infection; SARI = severe acute respiratory infection; ILI = influenza-like illness; ALRI = acute lower respiratory infection; PCR = polymerase chain reaction; NA = not available

RSV burden data

The RSV burden data among infants in LMICs (as one region) were obtained from our previously published RSV global burden estimates.[19] The data were available in the following age groups: <28 days, 1–<3 months, 3–<6 months, 6–<9 months and 9–<12 months; and by two outcomes: RSV-ALRI incidence rate in the community and RSV-ALRI hospitalisation rate. For the present study, we used the aggregated regional level percentage of RSV cases among infants <1y for each group as the model input (as shown in the table below).

Model input of RSV incidence data

Age Group	RSV-ALRI	RSV-ALRI hospitalisation
<1m	14.91	15.10
1-<3m	10.40	33.32
3-<6m	25.26	23.92
6-<9m	22.98	16.58
9-<12m	26.45	11.08

For each outcome, the percentage results add up to 100% across age groups.

RSV prophylactic coverage data

For monoclonal antibody immunisation, data on BCG and Hepatitis B vaccines coverage were included from the World Health Organization (WHO).[20] The average coverage between the two vaccines was calculated for each country. If coverage was missing for one vaccine for a country, then the coverage of the other vaccine was used.

As limited data were available on the maternal influenza vaccine coverage in LMICs, we used the WHO ANC4+ indicator for the maternal vaccine coverage, defined as the percentage of women aged 15–49 with a live birth who received antenatal care (ANC) four or more times.[21] WHO did not report a separate indicator for each of the ANC visits.

Efficacy data

We used the ResVax efficacy data from its phase 3 clinical trial results among third-trimester pregnant women: 39·4% (95% CI: 5·3–61·2) for medically significant RSV-ALRI and 44·4% (95% CI: 19·6–61·5) for RSV-ALRI hospitalisation by day 90 after birth.[22] We used the Nirsevimab efficacy data from its phase 2b clinical trial results among healthy preterm infants: 70·1% (95% CI: 52·3–81·2) for medically attended RSV-ALRI and 78·4% (95% CI: 51·9–80·3) for RSV-ALRI hospitalisation by day 150 after inoculation.[23]

Details on the candidate seasonal approaches

mAb

- Seasonal approach A: mAb being administered in each epidemic month
- Seasonal approach B: mAb being administered in each month if there are two or more epidemic months among that month and the following two months (i.e. three months in total), allowing advanced administration by up to one month
- Seasonal approach C: mAb being administered in each month if there are two or more epidemic months among that month and the following three months (i.e. four months in total), allowing advanced administration by up to two months
- Seasonal approach D: mAb being administered in each month if there are two or more epidemic months among that month and the following four months (i.e. five months in total), allowing advanced administration by up to three months

Maternal vaccine

- Seasonal approach A: maternal vaccine being administered to pregnant woman whose expected date of delivery is in an epidemic month
- Seasonal approach B: maternal vaccine being administered to pregnant woman if there are two or more epidemic months among the expected month of delivery and the following two months (i.e. three months in total), allowing advanced administration by up to one month

Mathematical modelling

Notation

For simplicity, we present here the calculations for a specific country and a specific RSV outcome.

Symbol	Definition	Values
Superscripts		
a	Age group (months)	1:<1mo, 2:1-<2mo, …., 12:11-<12mo
m	Month of the year	1:Jan, 2:Feb, …., 12:Dec
Subscripts		
c	Candidate approach	1:Seasonal A+mAbs, 2: Seasonal B+mAbs, 3: Seasonal C+mAbs, 4: Seasonal D+mAbs, 5: Year-round+mAbs, , 6: Seasonal A+mat vac, 7:Seasonal B+mat vac , 8:Year-round+ mat vac
b	Month of birth	1:Jan, 2:Feb, …., 12:Dec

Symbol	Definition
Z^a	Annual incidence of RSV outcome in age group *a*
APP^m	Annual average percentage of RSV activity in month *m*
$p^{a,m}$	Proportion of **monthly** incidence for each age group among **annual** incidence in <6m
e_c	Efficacy of prophylactic in candidate approach *c*
c_c	Coverage of prophylactic in candidate approach *c*
I_c^{max}	Calculating proportion of annual incidence in <6m that can be averted by an RSV prophylactic if assuming 100% efficacy and 100% coverage
I_c	Effectiveness of candidate approach *c* including coverage and efficacy
μ	Number of live births per month
N_c	Number of months treatment is given for candidate approach *c*
D_c	Per-dose effectiveness of candidate approach *c*
R_c	Relative effectiveness of candidate approach *c* compared to the year-round approach

Calculations related to the effectiveness and relative efficiency

We calculated the proportion of monthly of incidence for age group *a* among annual incidence in <6 month using the formula,

$$p^{a,m} = \frac{Z^a APP^m}{\sum_{a=1}^{6} \sum_{m=1}^{12} Z^a APP^m}$$

For each candidate approach *c,* we determined whether each month and age group pair *(a,m)* is protected by the prophylactic treatment. If *(a,m)* is protected then it is a "benefit group", if it is not protected then it is a "non-benefit group". Therefore, by defining an indicator function $\mathbf{1}_c(a,m) = 1$ if *(a,m)* is a benefit group and 0 otherwise, we calculated the effectiveness of a candidate approach (I_c) with coverage c_c and efficacy e_c through the formulae,

$$I_c^{max} = \sum_{a=1}^{6} \sum_{m=1}^{12} p^{a,m} \mathbf{1}_c(a,m)$$

$$I_c = I_c^{max}\boldsymbol{e_c c_c}$$

To determine the per-dose effectiveness for each candidate approach (D_c), we calculated the ratio of the effectiveness and the number of doses given, resulting in the formula,

$$D_c = \frac{I_c}{\mu N_c}$$

To determine the relative efficiency (*R*$_c$), we calculated the ratio of the per-dose effectiveness between each candidate approach and the year-round approach. That is,

$$\text{For c} = \{1, \ldots, 5\}, R_c = \frac{D_c}{D_5}, \text{ otherwise } R_c = \frac{D_c}{D_8}$$

Calculations related to RSV-ALRI hospitalisations by birth month

To calculate the proportion of RSV-ALRI hospitalisations in <3m by birth month, *b*, we used the formula,

$$p_b = \frac{\sum_{a=1}^{3} p^{a,[(b+a-1)\ mod\ 12]+1}}{\sum_{a=1}^{3}\sum_{m=1}^{12} p^{a,m}}$$

Where $(b + a - 1)\ mod\ 12$ is the value of $(b + a + 1)$ modulus 12.

Summary of the base case values and the values for sensitivity analyses

Variable	Base case	Sensitivity analyses
**Monoclonal antibodies**		
**RSV incidence**	Table on Page 6 of appendix[19]	—
**Duration of protection**	5 months[23]	—
**Coverage**	WHO BCG/HepB_BD[20]	100% (hypothetical)
**Efficacy against RSV-ALRI**	0.701 for each of the first 5 months, 0 thereafter[23]	0.861, 0.690, 0.552, 0.441 and 0.353 for the first 5 months, 0 thereafter (based on hypothetical decay rate of 0.8)
**Efficacy against RSV-ALRI hospitalisation**	0.784 for each of the first 5 months, 0 thereafter[23]	0.964, 0.771, 0.617, 0.494 and 0.395 for the first 5 months, 0 thereafter (based on hypothetical decay rate of 0.8)
**Maternal vaccine**		
**RSV incidence**	Table on Page 6 of appendix[19]	—
**Duration of protection**	3 months[22]	5 months[23]
**Coverage**	WHO ANC4+[21]	100% (hypothetical)
**Efficacy against RSV-ALRI**	0.394 for each of the first 3 months, 0 thereafter[22]	Scenario 1: 0.484, 0.388, 0.310 for the first 3 months, 0 thereafter (based on hypothetical decay rate of 0.8)<br>Scenario 2: 0.701 for each of the first 5 months, 0 thereafter (i.e. having mAb efficacy)[23]
**Efficacy against RSV-ALRI hospitalisation**	0.444 for each of the first 3 months, 0 thereafter[22]	Scenario 1: 0.546, 0.437, 0.349 for the first 3 months, 0 thereafter (based on hypothetical decay rate of 0.8)<br>Scenario 2: 0.784 for each of the first 5 months, 0 thereafter (i.e. having mAb efficacy)[23]

Supplementary tables of results

Table S1. Effectiveness and relative efficiency for each mAb candidate approach

Approach	Dose months	Effectiveness	Effectiveness if 100% coverage	Effectiveness ratio	Relative efficiency
**Outcome: RSV-ALRI**					
**Seasonal A**	4 (3–5)	20.1 (16.1–23.5)	24 (18.5–27.7)	0.44 (0.34–0.51)	1.14 (1.04–1.26)
**Seasonal B**	4 (3–5)	23.9 (20–29.2)	28.8 (23.3–33.4)	0.53 (0.43–0.62)	1.49 (1.25–1.6)
**Seasonal C**	5 (4–7)	32.6 (28–37.1)	38.6 (34.4–40.8)	0.71 (0.63–0.75)	1.57 (1.3–1.75)
**Seasonal D**	6 (5–8)	38.6 (34.6–42.9)	44.5 (43.5–46)	0.82 (0.8–0.85)	1.52 (1.26–1.8)
**Year-round**	12 (12–12)	47.6 (42–52.3)	54.2 (54.2–54.2)	Reference	Reference
**Outcome: RSV-ALRI hospitalisation**					
**Seasonal A**	4 (3–5)	26.7 (22.7–32.3)	32.3 (27–37.6)	0.49 (0.41–0.57)	1.3 (1.15–1.44)
**Seasonal B**	4 (3–5)	34 (28.4–39.1)	40.2 (34.8–44.9)	0.61 (0.53–0.68)	1.72 (1.39–2)
**Seasonal C**	5 (4–7)	42.5 (36.9–47.8)	49.9 (46.4–52.4)	0.75 (0.7–0.79)	1.65 (1.36–1.98)
**Seasonal D**	6.5 (5–8)	49.2 (44.2–53.8)	56 (54.1–57.6)	0.85 (0.82–0.87)	1.52 (1.29–1.9)
**Year-round**	12 (12–12)	58.1 (51.3–63.8)	66.2 (66.2–66.2)	Reference	Reference

Results are presented as median (IQR) among the included countries. Seasonal approach A administers mAb in each epidemic month, while seasonal approaches B–D begin administration of mAb 1, 2 and 3 months prior to the onset of the first epidemic month, respectively.

Table S2. Effectiveness and relative efficiency for each maternal vaccine candidate approach

Approach	Dose months	Effectiveness	Effectiveness if 100% coverage	Effectiveness ratio	Relative efficiency
**Outcome: RSV-ALRI**					
**Seasonal A**	4 (3–5)	6.8 (4.3–7.9)	8.5 (7.6–9.2)	0.68 (0.6–0.73)	1.85 (1.44–2.29)
**Seasonal B**	4 (3–5)	7.1 (4.8–8.2)	9 (8.1–9.6)	0.72 (0.64–0.76)	1.9 (1.53–2.55)
**Year-round**	12 (12–12)	10.3 (7–11.3)	12.6 (12.6–12.6)	Reference	Reference
**Outcome: RSV-ALRI hospitalisation**					
**Seasonal A**	4 (3–5)	11.2 (7.4–13.9)	15.1 (12.5–16.5)	0.64 (0.53–0.7)	1.71 (1.32–1.97)
**Seasonal B**	4 (3–5)	13.7 (9.1–15.9)	17.4 (15.9–18.1)	0.74 (0.67–0.77)	1.9 (1.56–2.62)
**Year-round**	12 (12–12)	19.4 (13.1–21.1)	23.6 (23.6–23.6)	Reference	Reference

Results are presented as median (IQR) among the included countries. Seasonal approach A is designed to protect infants born in each epidemic month, while seasonal approach B protects infants whose first three months of life include at least two RSV epidemic months.

Table S3. Effectiveness and relative efficiency for each mAb candidate approach among countries with ≤5 epidemic months, with monthly efficacy decay rate of 0.8

Approach	Dose months	Effectiveness	Effectiveness if 100% coverage	Effectiveness ratio	Relative efficiency
**Outcome: RSV-ALRI**					
**Seasonal A**	4 (3–5)	16.5 (14.7–19.6)	19.3 (16.9–22.1)	0.47 (0.41–0.54)	1.45 (1.34–1.63)
**Seasonal B**	4 (3–5)	19 (16.6–23.3)	23.1 (19.4–25.6)	0.56 (0.47–0.62)	1.74 (1.6–1.9)
**Seasonal C**	5 (4–6)	25.1 (20.8–28.7)	29.3 (26.4–30.4)	0.71 (0.64–0.74)	1.74 (1.55–1.93)
**Seasonal D**	6 (5–7)	28.7 (26.5–32.2)	32.9 (32.1–34.4)	0.8 (0.78–0.84)	1.65 (1.45–1.88)
**Year-round**	12 (12–12)	37.2 (32–39.8)	41.1 (41.1–41.1)	Reference	Reference
**Outcome: RSV-ALRI hospitalisation**					
**Seasonal A**	4 (3–5)	24 (19.8–27.9)	27.5 (22.6–31.1)	0.51 (0.42–0.58)	1.55 (1.45–1.75)
**Seasonal B**	4 (3–5)	28.8 (24.3–33.6)	32.9 (30.6–36.4)	0.62 (0.57–0.68)	2 (1.73–2.32)
**Seasonal C**	5 (4–6)	35.4 (31–39.9)	40.9 (38.5–42.1)	0.77 (0.72–0.79)	1.88 (1.61–2.17)
**Seasonal D**	6 (5–7)	39.3 (36.1–43)	44.9 (43.3–46.6)	0.84 (0.81–0.87)	1.67 (1.49–1.95)
**Year-round**	12 (12–12)	48.4 (41.6–51.8)	53.5 (53.5–53.5)	Reference	Reference

Results are presented as median (IQR) among the included countries. Seasonal approach A administers mAb in each epidemic month, while seasonal approaches B–D begin administration of mAb 1, 2 and 3 months prior to the onset of the first epidemic month, respectively.

Table S4. Effectiveness and relative efficiency for each maternal vaccine candidate approach in countries with ≤5 epidemic months, with monthly efficacy decay rate of 0.8

Approach	Dose months	Effectiveness	Effectiveness if 100% coverage	Effectiveness ratio	Relative efficiency
**Outcome: RSV-ALRI**					
**Seasonal A**	4 (3–5)	7.2 (5.8–8.2)	8.9 (8.2–9.4)	0.68 (0.63–0.72)	2.08 (1.83–2.54)
**Seasonal B**	4 (3–5)	7.2 (5.4–8.4)	9.1 (8.1–9.8)	0.7 (0.63–0.75)	2.23 (1.85–2.72)
**Year-round**	12 (12–12)	10.8 (9–11.5)	13 (13–13)	Reference	Reference
**Outcome: RSV-ALRI hospitalisation**					
**Seasonal A**	4 (3–5)	10.9 (8–13.4)	14.1 (12.1–15.3)	0.63 (0.54–0.68)	1.89 (1.73–2.18)
**Seasonal B**	4 (3–5)	13 (10–14.9)	16.4 (14.9–17)	0.73 (0.66–0.76)	2.25 (1.87–2.79)
**Year-round**	12 (12–12)	18.6 (15.6–19.9)	22.4 (22.4–22.4)	Reference	Reference

Results are presented as median (IQR) among the included countries. Seasonal approach A is designed to protect infants born in each epidemic month, while seasonal approach B protects infants whose first three months of life include at least two RSV epidemic months.

Table S5. Year-to-year variations in relative effectiveness and relative efficiency for each monoclonal antibodies candidate approach in countries with ≤5 epidemic months

Approach	Dose months	Effectiveness ratio	Relative efficiency
**Outcome: RSV-ALRI**			
**Seasonal A**	5 (4–5)	0.42 (0.34–0.51)	1.21 (1.01–1.42)
**Seasonal B**	5 (4–5)	0.56 (0.46–0.66)	1.59 (1.4–1.81)
**Seasonal C**	6 (5–6)	0.72 (0.63–0.83)	1.7 (1.45–1.84)
**Seasonal D**	7 (6–7)	0.84 (0.78–0.91)	1.59 (1.38–1.77)
**Year-round**	12 (12–12)	Reference	Reference
**Outcome: RSV-ALRI hospitalisation**			
**Seasonal A**	5 (4–5)	0.51 (0.42–0.6)	1.43 (1.23–1.71)
**Seasonal B**	5 (4–5)	0.64 (0.55–0.74)	1.86 (1.65–2.07)
**Seasonal C**	6 (5–6)	0.77 (0.71–0.86)	1.79 (1.59–2.01)
**Seasonal D**	7 (6–7)	0.87 (0.81–0.92)	1.61 (1.44–1.79)
**Year-round**	12 (12–12)	Reference	Reference

Results are presented as median (IQR) among all the study years. Seasonal approach A administers mAb in each epidemic month, while seasonal approaches B–D begin administration of mAb 1, 2 and 3 months prior to the onset of the first epidemic month, respectively.

Table S6. Year-to-year variations in the effectiveness and relative efficiency for each maternal vaccine candidate approach in countries with ≤5 epidemic months

Approach	Dose months	Effectiveness ratio	Relative efficiency
**Outcome: RSV-ALRI**			
**Seasonal A**	5 (4–5)	0.71 (0.62–0.81)	2.05 (1.62–2.28)
**Seasonal B**	5 (4–5)	0.76 (0.65–0.85)	2.09 (1.76–2.4)
**Year-round**	12 (12–12)	Reference	Reference
**Outcome: RSV-ALRI hospitalisation**			
**Seasonal A**	5 (4–5)	0.67 (0.53–0.8)	1.95 (1.57–2.25)
**Seasonal B**	5 (4–5)	0.77 (0.68–0.87)	2.15 (1.79–2.52)
**Year-round**	12 (12–12)	Reference	Reference

Results are presented as median (IQR) among all the study years. Seasonal approach A is designed to protect infants born in each epidemic month, while seasonal approach B protects infants whose first three months of life include at least two RSV epidemic months.

Table S7. Country-specific year-to-year variations in the effectiveness and relative efficiency for each monoclonal antibodies candidate approach

Country	Approach	N of years	Dosing months	Effectiveness	Relative efficiency
**Outcome: RSV-ALRI**					
Argentina	Seasonal A	8	3	16.5 (15.3–17.5)	1.39 (1.29–1.48)
Argentina	Seasonal B	8	3	22 (20.8–22.5)	1.86 (1.75–1.9)
Argentina	Seasonal C	8	4	32 (31.1–32.6)	2.03 (1.97–2.06)
Argentina	Seasonal D	8	5	38.4 (37.9–39.4)	1.94 (1.92–1.99)
Argentina	Year-round	8	12	47.4 (47.4–47.4)	Reference
Indonesia	Seasonal A	3	3	12.5 (11.3–14.7)	1.37 (1.23–1.61)
Indonesia	Seasonal B	3	3	16.4 (15.3–18)	1.79 (1.68–1.96)
Indonesia	Seasonal C	3	4	23.3 (22.9–23.5)	1.91 (1.87–1.92)
Indonesia	Seasonal D	3	5	29.6 (27.6–29.9)	1.94 (1.81–1.96)
Indonesia	Year-round	3	12	36.6 (36.6–36.6)	Reference
Jordan	Seasonal A	6	3	13.3 (12.8–13.6)	1.04 (1–1.07)
Jordan	Seasonal B	6	3	19.4 (18.4–19.7)	1.52 (1.44–1.55)
Jordan	Seasonal C	6	4	29.4 (28.1–29.9)	1.73 (1.65–1.76)
Jordan	Seasonal D	6	5	39.5 (38.7–40.1)	1.86 (1.82–1.89)
Jordan	Year-round	6	12	51 (51–51)	Reference
Cuba	Seasonal A	8	4	21.7 (18.7–26.6)	1.21 (1.04–1.49)
Cuba	Seasonal B	8	4	27.5 (25.5–34.5)	1.54 (1.43–1.93)
Cuba	Seasonal C	8	5	39.2 (35.2–41.8)	1.75 (1.57–1.87)
Cuba	Seasonal D	8	6	45.5 (41.3–47.6)	1.69 (1.54–1.77)
Cuba	Year-round	8	12	53.7 (53.7–53.7)	Reference
Madagascar	Seasonal A	6	4	15.3 (13.7–17.6)	1.21 (1.08–1.39)
Madagascar	Seasonal B	6	4	20.3 (19.1–22.8)	1.6 (1.51–1.8)
Madagascar	Seasonal C	6	5	29.2 (28.6–31)	1.85 (1.81–1.96)
Madagascar	Seasonal D	6	6	35.3 (34.1–35.7)	1.86 (1.8–1.88)
Madagascar	Year-round	6	12	38 (38–38)	Reference
South Africa	Seasonal A	4	4	16 (13.8–18.1)	1.27 (1.09–1.43)
South Africa	Seasonal B	4	4	20.6 (19.7–21.5)	1.63 (1.55–1.7)
South Africa	Seasonal C	4	5	26.8 (26–27.5)	1.69 (1.64–1.74)
South Africa	Seasonal D	4	6	31.3 (30.9–31.6)	1.65 (1.63–1.67)
South Africa	Year-round	4	12	38 (38–38)	Reference
Thailand	Seasonal A	4	4	23.4 (22.5–24.8)	1.31 (1.26–1.39)
Thailand	Seasonal B	4	4	29.7 (29.5–30.9)	1.66 (1.65–1.72)
Thailand	Seasonal C	4	5	39 (38.1–39.9)	1.74 (1.7–1.79)
Thailand	Seasonal D	4	6	44.9 (43.9–45.3)	1.67 (1.64–1.69)
Thailand	Year-round	4	12	53.7 (53.7–53.7)	Reference
Bolivia	Seasonal A	3	5	28 (20.8–30.8)	1.38 (1.03–1.52)
Bolivia	Seasonal B	3	5	36.5 (28–37.5)	1.79 (1.38–1.84)
Bolivia	Seasonal C	3	6	42.4 (34.9–42.4)	1.74 (1.43–1.74)
Bolivia	Seasonal D	3	7	43.9 (40.9–44.5)	1.54 (1.44–1.56)
Bolivia	Year-round	3	12	48.8 (48.8–48.8)	Reference
Ecuador	Seasonal A	6	5	19.9 (18–25.1)	1.1 (1–1.39)
Ecuador	Seasonal B	6	5	26.3 (24.9–26.6)	1.45 (1.38–1.47)

Country	Approach	N of years	Dosing months	Effectiveness	Relative efficiency
Ecuador	Seasonal C	6	6	34.1 (31.9–34.2)	1.57 (1.47–1.58)
Ecuador	Seasonal D	6	7	38 (35.5–39.4)	1.5 (1.4–1.56)
Ecuador	Year-round	6	12	43.4 (43.4–43.4)	Reference
Egypt	Seasonal A	6	5	21.2 (20.8–25.9)	1.02 (1–1.25)
Egypt	Seasonal B	6	5	30.7 (29.8–34.2)	1.48 (1.43–1.65)
Egypt	Seasonal C	6	6	40.8 (39.4–42.5)	1.64 (1.58–1.7)
Egypt	Seasonal D	6	7	45.6 (44.8–46.6)	1.57 (1.54–1.6)
Egypt	Year-round	6	12	49.9 (49.9–49.9)	Reference
Gambia	Seasonal A	5	5	21.8 (17.5–21.8)	1.02 (0.82–1.03)
Gambia	Seasonal B	5	4	24.6 (23.1–28.6)	1.45 (1.36–1.68)
Gambia	Seasonal C	5	5	32.2 (30.8–37.5)	1.51 (1.45–1.76)
Gambia	Seasonal D	5	7	43 (40.9–46.5)	1.45 (1.38–1.56)
Gambia	Year-round	5	12	51 (51–51)	Reference
Honduras	Seasonal A	8	5	26.7 (22.8–28.2)	1.36 (1.17–1.44)
Honduras	Seasonal B	8	5	31.6 (24.9–34.9)	1.62 (1.27–1.79)
Honduras	Seasonal C	8	6	37.3 (28.1–40)	1.59 (1.2–1.71)
Honduras	Seasonal D	8	7	40.5 (32.2–41.9)	1.48 (1.18–1.53)
Honduras	Year-round	8	12	46.9 (46.9–46.9)	Reference
Mexico	Seasonal A	6	5	24 (21.2–28.8)	1.09 (0.97–1.32)
Mexico	Seasonal B	6	6	33.4 (30.2–40.4)	1.27 (1.15–1.54)
Mexico	Seasonal C	6	7	38.4 (36.1–42.6)	1.25 (1.18–1.39)
Mexico	Seasonal D	6	8	43.4 (41.9–45.4)	1.24 (1.2–1.29)
Mexico	Year-round	6	12	52.6 (52.6–52.6)	Reference
Nicaragua	Seasonal A	8	5	25.9 (23.5–36.3)	1.17 (1.06–1.64)
Nicaragua	Seasonal B	8	5	37.3 (28–44.5)	1.68 (1.26–2.01)
Nicaragua	Seasonal C	8	6	46 (36.6–47.1)	1.73 (1.38–1.77)
Nicaragua	Seasonal D	8	7	47.9 (43.5–49.4)	1.55 (1.4–1.59)
Nicaragua	Year-round	8	12	53.1 (53.1–53.1)	Reference
Paraguay	Seasonal A	8	5	24 (18.6–27.9)	1.17 (0.91–1.36)
Paraguay	Seasonal B	8	5	31.7 (25.6–35)	1.54 (1.25–1.7)
Paraguay	Seasonal C	8	6	38.9 (31.7–41.9)	1.58 (1.29–1.7)
Paraguay	Seasonal D	8	7	41.9 (37.3–45.7)	1.45 (1.29–1.59)
Paraguay	Year-round	8	12	49.3 (49.3–49.3)	Reference
Costa Rica	Seasonal A	8	6	29 (24.6–36.8)	1.18 (1–1.49)
Costa Rica	Seasonal B	8	6	33.2 (29.2–40.1)	1.35 (1.18–1.63)
Costa Rica	Seasonal C	8	7	39.6 (33.7–42.3)	1.38 (1.17–1.47)
Costa Rica	Seasonal D	8	8	42.7 (37–45.5)	1.3 (1.13–1.38)
Costa Rica	Year-round	8	12	49.3 (49.3–49.3)	Reference
Dominican Rep	Seasonal A	3	6	37.5 (31.6–38.9)	1.69 (1.42–1.75)
Dominican Rep	Seasonal B	3	6	39.2 (37.5–39.4)	1.76 (1.69–1.77)
Dominican Rep	Seasonal C	3	7	41.1 (40.2–41.5)	1.59 (1.55–1.6)
Dominican Rep	Seasonal D	3	8	41.6 (40.6–42.6)	1.4 (1.37–1.44)
Dominican Rep	Year-round	3	12	44.5 (44.5–44.5)	Reference
El Salvador	Seasonal A	8	6	22.2 (18.9–33.6)	1.04 (0.88–1.57)
El Salvador	Seasonal B	8	6	28.1 (22.9–32)	1.31 (1.07–1.49)
El Salvador	Seasonal C	8	7	32.1 (29.2–35.5)	1.29 (1.17–1.42)

Country	Approach	N of years	Dosing months	Effectiveness	Relative efficiency
El Salvador	Seasonal D	8	8	35.3 (34.6–36)	1.24 (1.21–1.26)
El Salvador	Year-round	8	12	42.8 (42.8–42.8)	Reference
Guatemala	Seasonal A	8	6	19.9 (17.1–24.3)	1.08 (0.93–1.32)
Guatemala	Seasonal B	8	6	24.5 (22–26.6)	1.33 (1.19–1.44)
Guatemala	Seasonal C	8	7	28.8 (26.8–29.9)	1.34 (1.25–1.39)
Guatemala	Seasonal D	8	8	31.3 (28.8–32.8)	1.28 (1.17–1.34)
Guatemala	Year-round	8	12	36.9 (36.9–36.9)	Reference
Kenya	Seasonal A	4	6	29.2 (26.1–32.6)	1.14 (1.01–1.27)
Kenya	Seasonal B	4	5	22.2 (17.6–25.4)	1.04 (0.82–1.18)
Kenya	Seasonal C	4	7	30.5 (26.7–33.3)	1.01 (0.89–1.11)
Kenya	Seasonal D	4	8	34.1 (30.8–35.7)	0.99 (0.9–1.04)
Kenya	Year-round	4	12	51.5 (51.5–51.5)	Reference
Peru	Seasonal A	8	6	18.9 (18–22.8)	0.91 (0.86–1.09)
Peru	Seasonal B	8	6	24.8 (23–29.1)	1.19 (1.1–1.39)
Peru	Seasonal C	8	7	31.9 (30–33.9)	1.31 (1.23–1.39)
Peru	Seasonal D	8	8	36.4 (35.1–37.2)	1.31 (1.26–1.33)
Peru	Year-round	8	12	41.7 (41.7–41.7)	Reference
Philippines	Seasonal A	3	6	18 (17.5–19.5)	1.06 (1.03–1.15)
Philippines	Seasonal B	3	6	23.1 (22.1–24.8)	1.36 (1.3–1.46)
Philippines	Seasonal C	3	7	27.2 (26.4–28.4)	1.37 (1.33–1.44)
Philippines	Seasonal D	3	8	28.9 (28.6–30.2)	1.28 (1.27–1.34)
Philippines	Year-round	3	12	33.9 (33.9–33.9)	Reference
Bangladesh	Seasonal A	3	7	32.9 (28.9–33)	1.05 (0.92–1.05)
Bangladesh	Seasonal B	3	7	33.1 (31.7–33.2)	1.06 (1.01–1.06)
Bangladesh	Seasonal C	3	10	53.7 (48.2–53.7)	1.2 (1.08–1.2)
Bangladesh	Seasonal D	3	12	53.7 (53.7–53.7)	Reference
Bangladesh	Year-round	3	12	53.7 (53.7–53.7)	Reference
China	Seasonal A	3	7	32.4 (31–35.5)	1.05 (1.01–1.15)
China	Seasonal B	3	7	36.4 (35.7–38.9)	1.18 (1.16–1.26)
China	Seasonal C	3	8	44 (41.9–44.5)	1.25 (1.19–1.26)
China	Seasonal D	3	9	47.5 (45.6–48)	1.2 (1.15–1.21)
China	Year-round	3	12	52.9 (52.9–52.9)	Reference
Colombia	Seasonal A	8	8	27.3 (26.1–28.9)	0.91 (0.87–0.96)
Colombia	Seasonal B	8	9	32.9 (31.1–33.6)	0.97 (0.92–1)
Colombia	Seasonal C	8	10	38.4 (36.3–38.8)	1.02 (0.97–1.03)
Colombia	Seasonal D	8	12	45 (45–45)	Reference
Colombia	Year-round	8	12	45 (45–45)	Reference
Outcome: RSV-ALRI hospitalisation					
Argentina	Seasonal A	8	3	26.1 (24–27.6)	1.8 (1.66–1.91)
Argentina	Seasonal B	8	3	33.6 (32.6–34.1)	2.32 (2.25–2.35)
Argentina	Seasonal C	8	4	42.8 (41.8–43.5)	2.22 (2.16–2.25)
Argentina	Seasonal D	8	5	48 (47.3–49.6)	1.99 (1.96–2.05)
Argentina	Year-round	8	12	57.9 (57.9–57.9)	Reference
Indonesia	Seasonal A	3	3	18.8 (16.9–21.5)	1.69 (1.51–1.93)
Indonesia	Seasonal B	3	3	23.6 (23.4–24.1)	2.11 (2.1–2.16)
Indonesia	Seasonal C	3	4	31.9 (29.3–32.1)	2.14 (1.96–2.16)

Country	Approach	N of years	Dosing months	Effectiveness	Relative efficiency
Indonesia	Seasonal D	3	5	36.8 (32.8–37.9)	1.98 (1.76–2.04)
Indonesia	Year-round	3	12	44.7 (44.7–44.7)	Reference
Jordan	Seasonal A	6	3	19.8 (18.8–20.1)	1.27 (1.21–1.29)
Jordan	Seasonal B	6	3	30.7 (28.9–32)	1.98 (1.86–2.06)
Jordan	Seasonal C	6	4	43.2 (42.6–44.7)	2.08 (2.05–2.16)
Jordan	Seasonal D	6	5	51.7 (51.1–53.1)	1.99 (1.97–2.05)
Jordan	Year-round	6	12	62.2 (62.2–62.2)	Reference
Cuba	Seasonal A	8	4	30.9 (28.5–40.8)	1.42 (1.31–1.87)
Cuba	Seasonal B	8	4	41 (36.8–45.7)	1.88 (1.69–2.09)
Cuba	Seasonal C	8	5	50.5 (46.7–53.9)	1.85 (1.71–1.98)
Cuba	Seasonal D	8	6	56.8 (49.8–59.1)	1.73 (1.52–1.8)
Cuba	Year-round	8	12	65.5 (65.5–65.5)	Reference
Madagascar	Seasonal A	6	4	23.5 (21.7–26.2)	1.52 (1.4–1.7)
Madagascar	Seasonal B	6	4	31.5 (30.4–33)	2.04 (1.97–2.14)
Madagascar	Seasonal C	6	5	39.4 (39–39.5)	2.04 (2.02–2.05)
Madagascar	Seasonal D	6	6	44 (41.8–44.3)	1.9 (1.8–1.91)
Madagascar	Year-round	6	12	46.3 (46.3–46.3)	Reference
South Africa	Seasonal A	4	4	23.2 (20.5–25.4)	1.5 (1.33–1.64)
South Africa	Seasonal B	4	4	28.8 (28–29.5)	1.86 (1.81–1.91)
South Africa	Seasonal C	4	5	35.5 (34.9–36)	1.84 (1.81–1.86)
South Africa	Seasonal D	4	6	39.1 (37.6–40.8)	1.69 (1.62–1.76)
South Africa	Year-round	4	12	46.3 (46.3–46.3)	Reference
Thailand	Seasonal A	4	4	34 (33.4–35.7)	1.56 (1.53–1.64)
Thailand	Seasonal B	4	4	41.8 (40.8–43.2)	1.91 (1.87–1.98)
Thailand	Seasonal C	4	5	51.1 (49.4–52.1)	1.87 (1.81–1.91)
Thailand	Seasonal D	4	6	56 (54.8–56.3)	1.71 (1.67–1.72)
Thailand	Year-round	4	12	65.5 (65.5–65.5)	Reference
Bolivia	Seasonal A	3	5	40.3 (29.1–43.1)	1.62 (1.17–1.74)
Bolivia	Seasonal B	3	5	48.6 (38.9–49)	1.96 (1.57–1.98)
Bolivia	Seasonal C	3	6	53.1 (47–53.3)	1.78 (1.58–1.79)
Bolivia	Seasonal D	3	7	54.2 (52.4–54.9)	1.56 (1.51–1.58)
Bolivia	Year-round	3	12	59.6 (59.6–59.6)	Reference
Ecuador	Seasonal A	6	5	29.2 (26.3–32.8)	1.32 (1.19–1.49)
Ecuador	Seasonal B	6	5	36.7 (34.4–37.2)	1.66 (1.56–1.69)
Ecuador	Seasonal C	6	6	42.8 (41–44.4)	1.62 (1.55–1.68)
Ecuador	Seasonal D	6	7	47.5 (42.9–48.5)	1.54 (1.39–1.57)
Ecuador	Year-round	6	12	52.9 (52.9–52.9)	Reference
Egypt	Seasonal A	6	5	32.9 (31.6–37.7)	1.3 (1.24–1.48)
Egypt	Seasonal B	6	5	44.2 (42.3–46)	1.74 (1.67–1.81)
Egypt	Seasonal C	6	6	52.6 (51.4–54.1)	1.73 (1.69–1.78)
Egypt	Seasonal D	6	7	56.9 (55.6–57.6)	1.6 (1.57–1.62)
Egypt	Year-round	6	12	60.9 (60.9–60.9)	Reference
Gambia	Seasonal A	5	5	29.3 (24.2–32)	1.13 (0.93–1.23)
Gambia	Seasonal B	5	4	34.3 (34.1–41.2)	1.65 (1.65–1.99)
Gambia	Seasonal C	5	5	45.1 (41.8–50)	1.74 (1.61–1.93)
Gambia	Seasonal D	5	7	54.8 (52.3–57.8)	1.51 (1.44–1.59)

Country	Approach	N of years	Dosing months	Effectiveness	Relative efficiency
Gambia	Year-round	5	12	62.2 (62.2–62.2)	Reference
Honduras	Seasonal A	8	5	36.1 (30.5–40)	1.51 (1.28–1.68)
Honduras	Seasonal B	8	5	42.5 (32.8–45.8)	1.78 (1.37–1.92)
Honduras	Seasonal C	8	6	47.6 (36.8–50.3)	1.66 (1.29–1.76)
Honduras	Seasonal D	8	7	50.3 (40.9–52.2)	1.51 (1.22–1.56)
Honduras	Year-round	8	12	57.2 (57.2–57.2)	Reference
Mexico	Seasonal A	6	5	29.8 (25.3–33.8)	1.11 (0.95–1.26)
Mexico	Seasonal B	6	6	43.4 (39–50.7)	1.35 (1.22–1.58)
Mexico	Seasonal C	6	7	50.4 (46.7–53.3)	1.35 (1.25–1.42)
Mexico	Seasonal D	6	8	55.6 (53.3–56.4)	1.3 (1.24–1.32)
Mexico	Year-round	6	12	64.2 (64.2–64.2)	Reference
Nicaragua	Seasonal A	8	5	39.4 (31.2–50.8)	1.46 (1.15–1.88)
Nicaragua	Seasonal B	8	5	50.4 (38.5–56.4)	1.87 (1.42–2.09)
Nicaragua	Seasonal C	8	6	57.8 (48.2–59.8)	1.78 (1.49–1.84)
Nicaragua	Seasonal D	8	7	60 (54.8–61.4)	1.59 (1.45–1.62)
Nicaragua	Year-round	8	12	64.9 (64.9–64.9)	Reference
Paraguay	Seasonal A	8	5	33.9 (28.1–40.7)	1.35 (1.12–1.62)
Paraguay	Seasonal B	8	5	43 (34.2–46)	1.71 (1.36–1.83)
Paraguay	Seasonal C	8	6	48.7 (41.2–53.4)	1.62 (1.37–1.77)
Paraguay	Seasonal D	8	7	51.9 (47.1–56.7)	1.48 (1.34–1.61)
Paraguay	Year-round	8	12	60.2 (60.2–60.2)	Reference
Costa Rica	Seasonal A	8	6	37.7 (31.4–48.3)	1.25 (1.04–1.6)
Costa Rica	Seasonal B	8	6	44.6 (36.6–48.5)	1.48 (1.21–1.61)
Costa Rica	Seasonal C	8	7	49.5 (40.6–53.6)	1.41 (1.15–1.53)
Costa Rica	Seasonal D	8	8	51.9 (44–56.6)	1.29 (1.1–1.41)
Costa Rica	Year-round	8	12	60.2 (60.2–60.2)	Reference
Dominican Rep	Seasonal A	3	6	47.9 (42.9–49.2)	1.76 (1.58–1.81)
Dominican Rep	Seasonal B	3	6	48.2 (47.6–48.8)	1.78 (1.76–1.8)
Dominican Rep	Seasonal C	3	7	50.6 (49.5–51.3)	1.6 (1.56–1.62)
Dominican Rep	Seasonal D	3	8	50.9 (49.8–52.1)	1.41 (1.38–1.44)
Dominican Rep	Year-round	3	12	54.3 (54.3–54.3)	Reference
El Salvador	Seasonal A	8	6	31.9 (25.4–42.6)	1.22 (0.97–1.63)
El Salvador	Seasonal B	8	6	36.5 (31.7–42)	1.4 (1.21–1.61)
El Salvador	Seasonal C	8	7	40.2 (38.3–43.3)	1.32 (1.25–1.42)
El Salvador	Seasonal D	8	8	43.5 (43.2–43.9)	1.25 (1.24–1.26)
El Salvador	Year-round	8	12	52.3 (52.3–52.3)	Reference
Guatemala	Seasonal A	8	6	27.1 (24.4–32.5)	1.2 (1.09–1.44)
Guatemala	Seasonal B	8	6	32.7 (30.1–34)	1.45 (1.34–1.51)
Guatemala	Seasonal C	8	7	36.7 (33.7–38.1)	1.4 (1.28–1.45)
Guatemala	Seasonal D	8	8	39.2 (35.7–40.8)	1.3 (1.19–1.36)
Guatemala	Year-round	8	12	45 (45–45)	Reference
Kenya	Seasonal A	4	6	32.7 (28.5–38.4)	1.04 (0.91–1.22)
Kenya	Seasonal B	4	5	23.8 (18.5–27.6)	0.91 (0.71–1.05)
Kenya	Seasonal C	4	7	36.7 (31.3–40.5)	1 (0.85–1.11)
Kenya	Seasonal D	4	8	41.9 (36.8–44.2)	1 (0.88–1.06)
Kenya	Year-round	4	12	62.9 (62.9–62.9)	Reference

Country	Approach	N of years	Dosing months	Effectiveness	Relative efficiency
**Peru**	**Seasonal A**	8	6	26.8 (24.8–32.2)	1.05 (0.97–1.26)
**Peru**	**Seasonal B**	8	6	34.8 (32.3–38.1)	1.36 (1.27–1.5)
**Peru**	**Seasonal C**	8	7	41.2 (39.4–42.6)	1.39 (1.33–1.43)
**Peru**	**Seasonal D**	8	8	44.9 (43.2–46)	1.32 (1.27–1.35)
**Peru**	**Year-round**	8	12	51 (51–51)	Reference
**Philippines**	**Seasonal A**	3	6	25.5 (24.3–27.2)	1.23 (1.18–1.32)
**Philippines**	**Seasonal B**	3	6	29.9 (28.8–31.9)	1.44 (1.39–1.54)
**Philippines**	**Seasonal C**	3	7	33.6 (33–35.3)	1.39 (1.37–1.46)
**Philippines**	**Seasonal D**	3	8	35.6 (35.4–37)	1.29 (1.28–1.34)
**Philippines**	**Year-round**	3	12	41.4 (41.4–41.4)	Reference
**Bangladesh**	**Seasonal A**	3	7	39.6 (37.1–41.2)	1.04 (0.97–1.08)
**Bangladesh**	**Seasonal B**	3	7	44.1 (42.1–46.6)	1.15 (1.1–1.22)
**Bangladesh**	**Seasonal C**	3	10	65.5 (60.3–65.5)	1.2 (1.1–1.2)
**Bangladesh**	**Seasonal D**	3	12	65.5 (65.5–65.5)	Reference
**Bangladesh**	**Year-round**	3	12	65.5 (65.5–65.5)	Reference
**China**	**Seasonal A**	3	7	41.2 (41.1–45.3)	1.09 (1.09–1.2)
**China**	**Seasonal B**	3	7	49 (46.5–50.6)	1.3 (1.24–1.34)
**China**	**Seasonal C**	3	8	55.7 (52.4–56)	1.29 (1.22–1.3)
**China**	**Seasonal D**	3	9	58.4 (56–59.4)	1.21 (1.16–1.23)
**China**	**Year-round**	3	12	64.5 (64.5–64.5)	Reference
**Colombia**	**Seasonal A**	8	8	35.2 (33.7–36.3)	0.96 (0.92–0.99)
**Colombia**	**Seasonal B**	8	9	42.4 (39.9–43.6)	1.03 (0.97–1.06)
**Colombia**	**Seasonal C**	8	10	47.9 (46.4–48.6)	1.05 (1.01–1.06)
**Colombia**	**Seasonal D**	8	12	54.9 (54.9–54.9)	Reference
**Colombia**	**Year-round**	8	12	54.9 (54.9–54.9)	Reference

Countries are arranged by the duration of RSV epidemics from short (more seasonal) to long (less seasonal). Seasonal approach A administers mAb in each epidemic month, while seasonal approaches B–D begin administration of mAb 1, 2 and 3 months prior to the onset of the first epidemic month, respectively.

Table S8. Country-specific year-to-year variations in the effectiveness and relative efficiency for each maternal vaccine candidate approach

Country	Approach	N of years	Dosing months	Effectiveness	Relative efficiency
**Outcome: RSV-ALRI**					
Argentina	Seasonal A	8	3	7.8 (7.6–8)	2.77 (2.69–2.82)
Argentina	Seasonal B	8	3	7.7 (7.1–7.8)	2.71 (2.52–2.76)
Argentina	Year-round	8	12	11.3 (11.3–11.3)	Reference
Indonesia	Seasonal A	3	3	6.5 (6.1–6.6)	2.47 (2.33–2.5)
Indonesia	Seasonal B	3	3	7 (5.5–7.6)	2.67 (2.09–2.87)
Indonesia	Year-round	3	12	10.5 (10.5–10.5)	Reference
Jordan	Seasonal A	6	3	6.8 (6.4–7.2)	2.29 (2.15–2.41)
Jordan	Seasonal B	6	3	8.2 (7.5–8.8)	2.76 (2.53–2.97)
Jordan	Year-round	6	12	11.9 (11.9–11.9)	Reference
Cuba	Seasonal A	8	4	8.9 (7.7–9.5)	2.16 (1.88–2.31)
Cuba	Seasonal B	8	4	9.4 (7.2–10)	2.28 (1.75–2.42)
Cuba	Year-round	8	12	12.3 (12.3–12.3)	Reference
Madagascar	Seasonal A	6	4	5.1 (4.9–5.3)	2.36 (2.29–2.48)
Madagascar	Seasonal B	6	4	5.7 (5.3–5.9)	2.65 (2.45–2.76)
Madagascar	Year-round	6	12	6.4 (6.4–6.4)	Reference
South Africa	Seasonal A	4	4	6.6 (6.3–6.9)	2.07 (1.99–2.16)
South Africa	Seasonal B	4	4	6.8 (6.4–7.3)	2.15 (2–2.31)
South Africa	Year-round	4	12	9.5 (9.5–9.5)	Reference
Thailand	Seasonal A	4	4	8.2 (8–8.5)	2.15 (2.09–2.23)
Thailand	Seasonal B	4	4	8.2 (7.8–8.4)	2.13 (2.04–2.21)
Thailand	Year-round	4	12	11.5 (11.5–11.5)	Reference
Bolivia	Seasonal A	3	5	9.6 (7.7–9.7)	2.14 (1.71–2.15)
Bolivia	Seasonal B	3	5	8.4 (8.2–8.9)	1.88 (1.84–1.99)
Bolivia	Year-round	3	12	10.8 (10.8–10.8)	Reference
Ecuador	Seasonal A	6	5	7.7 (7.2–7.9)	1.84 (1.73–1.88)
Ecuador	Seasonal B	6	5	8.2 (6.4–8.6)	1.96 (1.53–2.06)
Ecuador	Year-round	6	12	10 (10–10)	Reference
Egypt	Seasonal A	6	5	8.5 (8.1–8.8)	1.96 (1.86–2.02)
Egypt	Seasonal B	6	5	9.3 (8.7–9.5)	2.13 (2.01–2.17)
Egypt	Year-round	6	12	10.4 (10.4–10.4)	Reference
Gambia	Seasonal A	5	5	6.2 (6.1–7.5)	1.53 (1.5–1.83)
Gambia	Seasonal B	5	4	7.4 (7.3–7.6)	2.26 (2.25–2.34)
Gambia	Year-round	5	12	9.8 (9.8–9.8)	Reference
Honduras	Seasonal A	8	5	9 (6.9–10)	1.93 (1.48–2.13)
Honduras	Seasonal B	8	5	8.7 (6.4–9.3)	1.87 (1.36–1.99)
Honduras	Year-round	8	12	11.2 (11.2–11.2)	Reference
Mexico	Seasonal A	6	5	6.9 (6.1–7.6)	1.4 (1.23–1.53)
Mexico	Seasonal B	6	6	9.3 (8.6–9.4)	1.56 (1.45–1.59)
Mexico	Year-round	6	12	11.9 (11.9–11.9)	Reference
Nicaragua	Seasonal A	8	5	9.6 (7.1–10.3)	2.08 (1.54–2.23)
Nicaragua	Seasonal B	8	5	9.2 (7.6–9.9)	2 (1.64–2.15)
Nicaragua	Year-round	8	12	11.1 (11.1–11.1)	Reference

Country	Approach	N of years	Dosing months	Effectiveness	Relative efficiency
Paraguay	Seasonal A	8	5	7.6 (6–8.4)	1.86 (1.46–2.05)
Paraguay	Seasonal B	8	5	7.5 (6.5–8.6)	1.85 (1.58–2.1)
Paraguay	Year-round	8	12	9.8 (9.8–9.8)	Reference
Costa Rica	Seasonal A	8	6	9.2 (7.8–10.1)	1.5 (1.26–1.65)
Costa Rica	Seasonal B	8	6	8.8 (7–11)	1.43 (1.13–1.78)
Costa Rica	Year-round	8	12	12.3 (12.3–12.3)	Reference
Dominican Rep	Seasonal A	3	6	11.3 (11.2–11.3)	1.88 (1.86–1.88)
Dominican Rep	Seasonal B	3	6	10.5 (9.7–11.1)	1.74 (1.61–1.84)
Dominican Rep	Year-round	3	12	12 (12–12)	Reference
El Salvador	Seasonal A	8	6	7.7 (6.7–8.8)	1.49 (1.3–1.7)
El Salvador	Seasonal B	8	6	7.4 (6.7–7.7)	1.44 (1.3–1.49)
El Salvador	Year-round	8	12	10.3 (10.3–10.3)	Reference
Guatemala	Seasonal A	8	6	8.6 (7.9–8.7)	1.58 (1.45–1.61)
Guatemala	Seasonal B	8	6	8.4 (7–9.3)	1.54 (1.29–1.72)
Guatemala	Year-round	8	12	10.9 (10.9–10.9)	Reference
Kenya	Seasonal A	4	6	2.4 (2.1–2.8)	0.81 (0.7–0.93)
Kenya	Seasonal B	4	5	1.6 (1.4–1.7)	0.63 (0.55–0.69)
Kenya	Year-round	4	12	5.9 (5.9–5.9)	Reference
Peru	Seasonal A	8	6	8.8 (8.2–9.4)	1.49 (1.38–1.59)
Peru	Seasonal B	8	6	9.5 (8.7–10.1)	1.6 (1.46–1.7)
Peru	Year-round	8	12	11.9 (11.9–11.9)	Reference
Philippines	Seasonal A	3	6	8.4 (8.1–8.9)	1.55 (1.49–1.64)
Philippines	Seasonal B	3	6	8.5 (8.4–9)	1.55 (1.54–1.65)
Philippines	Year-round	3	12	10.9 (10.9–10.9)	Reference
Bangladesh	Seasonal A	3	7	3.4 (3.1–3.8)	1.25 (1.13–1.4)
Bangladesh	Seasonal B	3	7	3.8 (3.4–4.2)	1.39 (1.23–1.53)
Bangladesh	Year-round	3	12	4.7 (4.7–4.7)	Reference
China	Seasonal A	3	7	NA (NA–NA)	1.41 (1.31–1.42)
China	Seasonal B	3	7	NA (NA–NA)	1.41 (1.32–1.47)
China	Year-round	3	12	NA (NA–NA)	Reference
Colombia	Seasonal A	8	8	8.5 (8–8.6)	1.12 (1.05–1.13)
Colombia	Seasonal B	8	9	9.6 (9.4–9.7)	1.12 (1.1–1.15)
Colombia	Year-round	8	12	11.3 (11.3–11.3)	Reference
**Outcome: RSV–ALRI hospitalisation**					
Argentina	Seasonal A	8	3	13.9 (13–14.4)	2.61 (2.46–2.72)
Argentina	Seasonal B	8	3	15 (14.8–16.1)	2.83 (2.79–3.02)
Argentina	Year-round	8	12	21.2 (21.2–21.2)	Reference
Indonesia	Seasonal A	3	3	11.6 (10.9–12)	2.36 (2.21–2.43)
Indonesia	Seasonal B	3	3	13.9 (11.4–14.5)	2.81 (2.3–2.94)
Indonesia	Year-round	3	12	19.7 (19.7–19.7)	Reference
Jordan	Seasonal A	6	3	10.5 (10.4–10.7)	1.89 (1.86–1.92)
Jordan	Seasonal B	6	3	15.5 (14.8–15.7)	2.77 (2.65–2.82)
Jordan	Year-round	6	12	22.3 (22.3–22.3)	Reference
Cuba	Seasonal A	8	4	15 (13.7–18.5)	1.95 (1.78–2.39)
Cuba	Seasonal B	8	4	18.1 (14.9–19.5)	2.35 (1.93–2.53)
Cuba	Year-round	8	12	23.1 (23.1–23.1)	Reference

Country	Approach	N of years	Dosing months	Effectiveness	Relative efficiency
Madagascar	Seasonal A	6	4	8.5 (8.1–9.6)	2.12 (2.02–2.39)
Madagascar	Seasonal B	6	4	10.9 (10.3–11.1)	2.72 (2.56–2.76)
Madagascar	Year-round	6	12	12.1 (12.1–12.1)	Reference
South Africa	Seasonal A	4	4	11.7 (11.2–12.1)	1.97 (1.88–2.04)
South Africa	Seasonal B	4	4	13.2 (12.7–13.7)	2.22 (2.13–2.31)
South Africa	Year-round	4	12	17.9 (17.9–17.9)	Reference
Thailand	Seasonal A	4	4	14.7 (14.5–15.3)	2.06 (2.03–2.14)
Thailand	Seasonal B	4	4	16.2 (15.7–16.5)	2.26 (2.2–2.3)
Thailand	Year-round	4	12	21.5 (21.5–21.5)	Reference
Bolivia	Seasonal A	3	5	17.5 (12.9–17.9)	2.08 (1.54–2.13)
Bolivia	Seasonal B	3	5	17.2 (15.6–17.8)	2.05 (1.85–2.11)
Bolivia	Year-round	3	12	20.2 (20.2–20.2)	Reference
Ecuador	Seasonal A	6	5	13.6 (12.7–13.9)	1.74 (1.63–1.78)
Ecuador	Seasonal B	6	5	15.4 (13–16.2)	1.96 (1.66–2.07)
Ecuador	Year-round	6	12	18.8 (18.8–18.8)	Reference
Egypt	Seasonal A	6	5	15.2 (14.2–15.9)	1.86 (1.75–1.95)
Egypt	Seasonal B	6	5	17.2 (16.6–17.9)	2.11 (2.03–2.19)
Egypt	Year-round	6	12	19.6 (19.6–19.6)	Reference
Gambia	Seasonal A	5	5	10.6 (9.8–12.9)	1.39 (1.28–1.69)
Gambia	Seasonal B	5	4	13.5 (12.5–14.9)	2.2 (2.04–2.43)
Gambia	Year-round	5	12	18.3 (18.3–18.3)	Reference
Honduras	Seasonal A	8	5	16.4 (12.4–18.2)	1.87 (1.41–2.08)
Honduras	Seasonal B	8	5	17.2 (12.9–18.3)	1.97 (1.47–2.09)
Honduras	Year-round	8	12	21 (21–21)	Reference
Mexico	Seasonal A	6	5	11.6 (10.1–12.4)	1.25 (1.09–1.34)
Mexico	Seasonal B	6	6	16.9 (15.4–18.1)	1.52 (1.38–1.62)
Mexico	Year-round	6	12	22.3 (22.3–22.3)	Reference
Nicaragua	Seasonal A	8	5	17.3 (12.8–19.6)	2 (1.48–2.26)
Nicaragua	Seasonal B	8	5	17.9 (14.9–18.9)	2.07 (1.72–2.19)
Nicaragua	Year-round	8	12	20.8 (20.8–20.8)	Reference
Paraguay	Seasonal A	8	5	14 (10.5–15.1)	1.83 (1.37–1.97)
Paraguay	Seasonal B	8	5	14.2 (12–16.5)	1.86 (1.57–2.16)
Paraguay	Year-round	8	12	18.4 (18.4–18.4)	Reference
Costa Rica	Seasonal A	8	6	17.1 (13.9–18.8)	1.48 (1.2–1.63)
Costa Rica	Seasonal B	8	6	17.9 (13.5–20.6)	1.55 (1.17–1.79)
Costa Rica	Year-round	8	12	23.1 (23.1–23.1)	Reference
Dominican Rep	Seasonal A	3	6	21.3 (21.1–21.4)	1.89 (1.87–1.9)
Dominican Rep	Seasonal B	3	6	20.6 (19.7–21.2)	1.83 (1.75–1.88)
Dominican Rep	Year-round	3	12	22.6 (22.6–22.6)	Reference
El Salvador	Seasonal A	8	6	14.5 (11.6–17.2)	1.5 (1.2–1.78)
El Salvador	Seasonal B	8	6	14.3 (13.9–14.5)	1.47 (1.44–1.49)
El Salvador	Year-round	8	12	19.4 (19.4–19.4)	Reference
Guatemala	Seasonal A	8	6	15.6 (14–16.1)	1.53 (1.38–1.58)
Guatemala	Seasonal B	8	6	16.3 (14.1–17.3)	1.6 (1.38–1.7)
Guatemala	Year-round	8	12	20.4 (20.4–20.4)	Reference
Kenya	Seasonal A	4	6	4.8 (4–5.9)	0.86 (0.73–1.05)

Country	Approach	N of years	Dosing months	Effectiveness	Relative efficiency
**Kenya**	**Seasonal B**	4	5	3.2 (2.6–3.5)	0.68 (0.56–0.76)
**Kenya**	**Year-round**	4	12	11.1 (11.1–11.1)	Reference
**Peru**	**Seasonal A**	8	6	15.4 (14–17.7)	1.38 (1.26–1.58)
**Peru**	**Seasonal B**	8	6	18.2 (16.8–18.5)	1.63 (1.5–1.66)
**Peru**	**Year-round**	8	12	22.3 (22.3–22.3)	Reference
**Philippines**	**Seasonal A**	3	6	15.6 (14.8–16.5)	1.53 (1.45–1.62)
**Philippines**	**Seasonal B**	3	6	16.1 (15.7–17.1)	1.57 (1.54–1.67)
**Philippines**	**Year-round**	3	12	20.5 (20.5–20.5)	Reference
**Bangladesh**	**Seasonal A**	3	7	6.4 (5.2–7)	1.24 (1.01–1.36)
**Bangladesh**	**Seasonal B**	3	7	6.5 (6.4–7.5)	1.27 (1.24–1.47)
**Bangladesh**	**Year-round**	3	12	8.8 (8.8–8.8)	Reference
**China**	**Seasonal A**	3	7	NA	1.35 (1.26–1.38)
**China**	**Seasonal B**	3	7	NA	1.44 (1.34–1.48)
**China**	**Year-round**	3	12	NA	Reference
**Colombia**	**Seasonal A**	8	8	15.5 (14–15.6)	1.09 (0.99–1.1)
**Colombia**	**Seasonal B**	8	9	17.8 (17.3–18.1)	1.12 (1.09–1.14)
**Colombia**	**Year-round**	8	12	21.3 (21.3–21.3)	Reference

Countries are arranged by the duration of RSV epidemics from short (more seasonal) to long (less seasonal). Seasonal approach A is designed to protect infants born in each epidemic month, while seasonal approach B protects infants whose first three months of life include at least two RSV epidemic months.

Table S9. Year-to-year variations in relative effectiveness and relative efficiency for each monoclonal antibodies candidate approach in countries with ≤5 epidemic months, with a monthly efficacy decay rate of 0.8

Approach	Dose months	Effectiveness ratio	Relative efficiency
**Outcome: RSV-ALRI**			
**Seasonal A**	5 (4–5)	0.5 (0.44–0.6)	1.48 (1.29–1.69)
**Seasonal B**	5 (4–5)	0.62 (0.5–0.71)	1.78 (1.58–1.93)
**Seasonal C**	6 (5–6)	0.73 (0.66–0.83)	1.71 (1.49–1.9)
**Seasonal D**	7 (6–7)	0.84 (0.77–0.91)	1.56 (1.42–1.72)
**Year-round**	12 (12–12)	Reference	Reference
**Outcome: RSV-ALRI hospitalisation**			
**Seasonal A**	5 (4–5)	0.57 (0.47–0.67)	1.63 (1.38–1.93)
**Seasonal B**	5 (4–5)	0.68 (0.6–0.79)	1.95 (1.67–2.18)
**Seasonal C**	6 (5–6)	0.8 (0.73–0.87)	1.81 (1.62–2.02)
**Seasonal D**	7 (6–7)	0.87 (0.81–0.92)	1.59 (1.44–1.8)
**Year-round**	12 (12–12)	Reference	Reference

Results are presented as median (IQR) among all the study years. Seasonal approach A administers mAb in each epidemic month, while seasonal approaches B–D begin administration of mAb 1, 2 and 3 months prior to the onset of the first epidemic month, respectively.

Table S10. Year-to-year variations in relative effectiveness and relative efficiency for each maternal vaccine candidate approach in countries with ≤5 epidemic months with a monthly efficacy decay rate of 0.8

Approach	Dose months	Effectiveness ratio	Relative efficiency
**Outcome: RSV-ALRI**			
**Seasonal A**	5 (4–5)	0.73 (0.66–0.84)	2.09 (1.66–2.36)
**Seasonal B**	5 (4–5)	0.76 (0.64–0.86)	2.09 (1.72–2.36)
**Year-round**	12 (12–12)	Reference	Reference
**Outcome: RSV-ALRI hospitalisation**			
**Seasonal A**	5 (4–5)	0.69 (0.57–0.8)	2.01 (1.59–2.25)
**Seasonal B**	5 (4–5)	0.77 (0.68–0.87)	2.14 (1.74–2.53)
**Year-round**	12 (12–12)	Reference	Reference

Results are presented as median (IQR) among all the study years. Seasonal approach A is designed to protect infants born in each epidemic month, while seasonal approach B protects infants whose first three months of life include at least two RSV epidemic months.

Supplementary figures

Figure S1. Schematic figure showing the workflow of the study

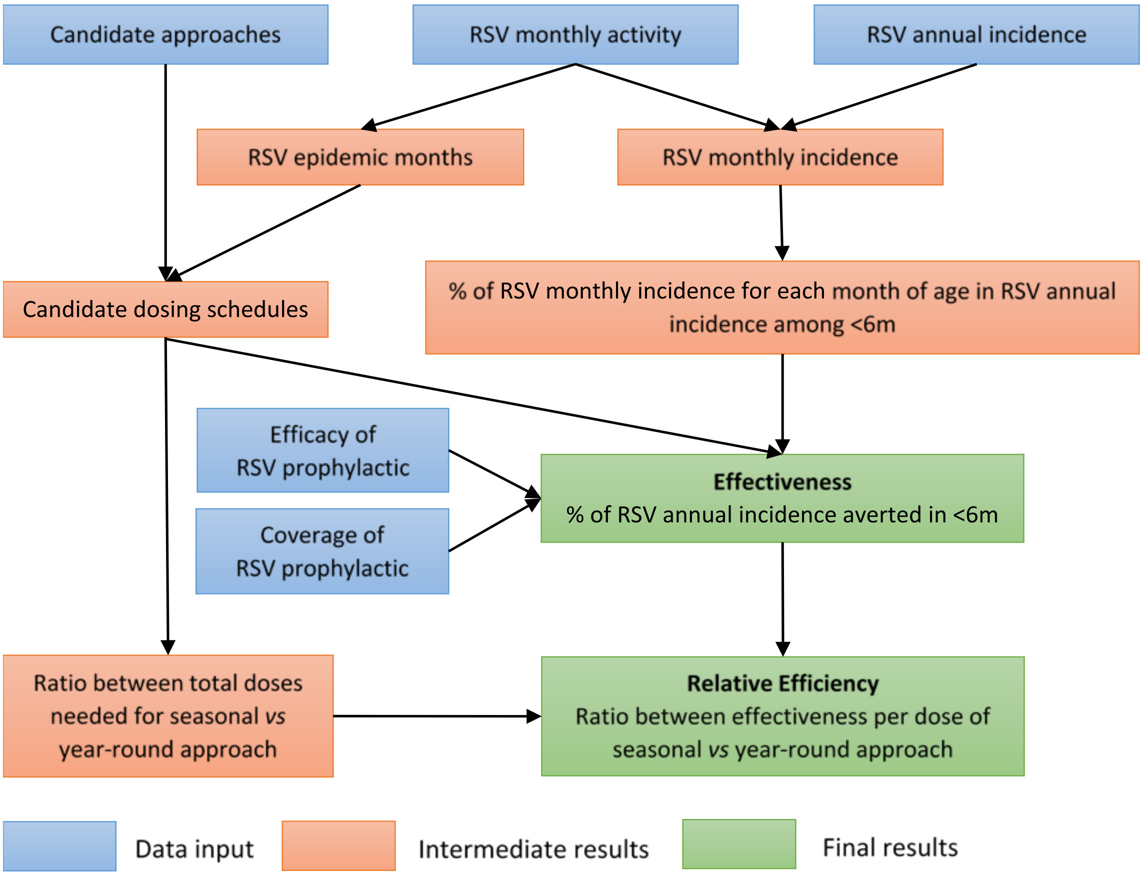

Figure S2. RSV activity data availability in LMICs

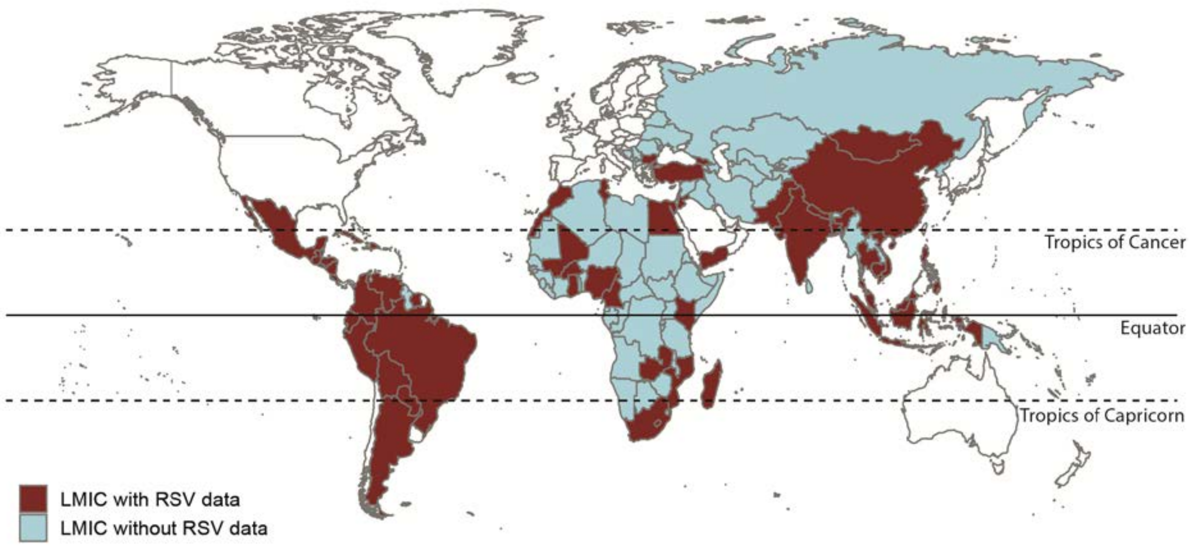

LMIC: low and middle income countries. List of LMICs from the 2019 World Bank Income Classification.[1]

Figure S3. Month-by-month activity of RSV in LMICs.

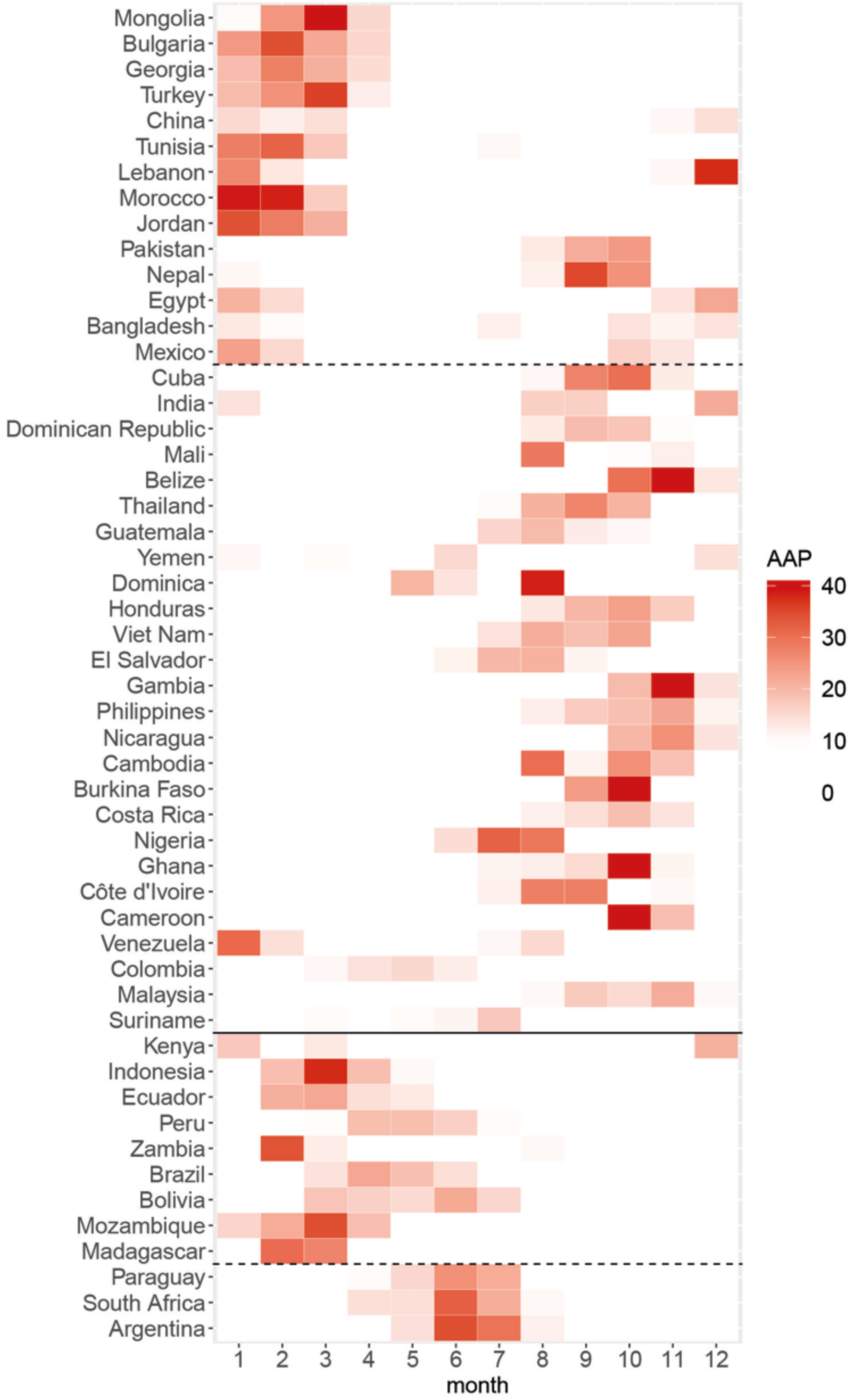

AAP: annual average percentage. LMICs are arranged by latitude. The solid line denotes the equator and the dashed lines denote tropics of Cancer and Capricorn. The months 1 to 12 represent January to December.

Figure S4. Month-by-month activity of RSV in LMICs.

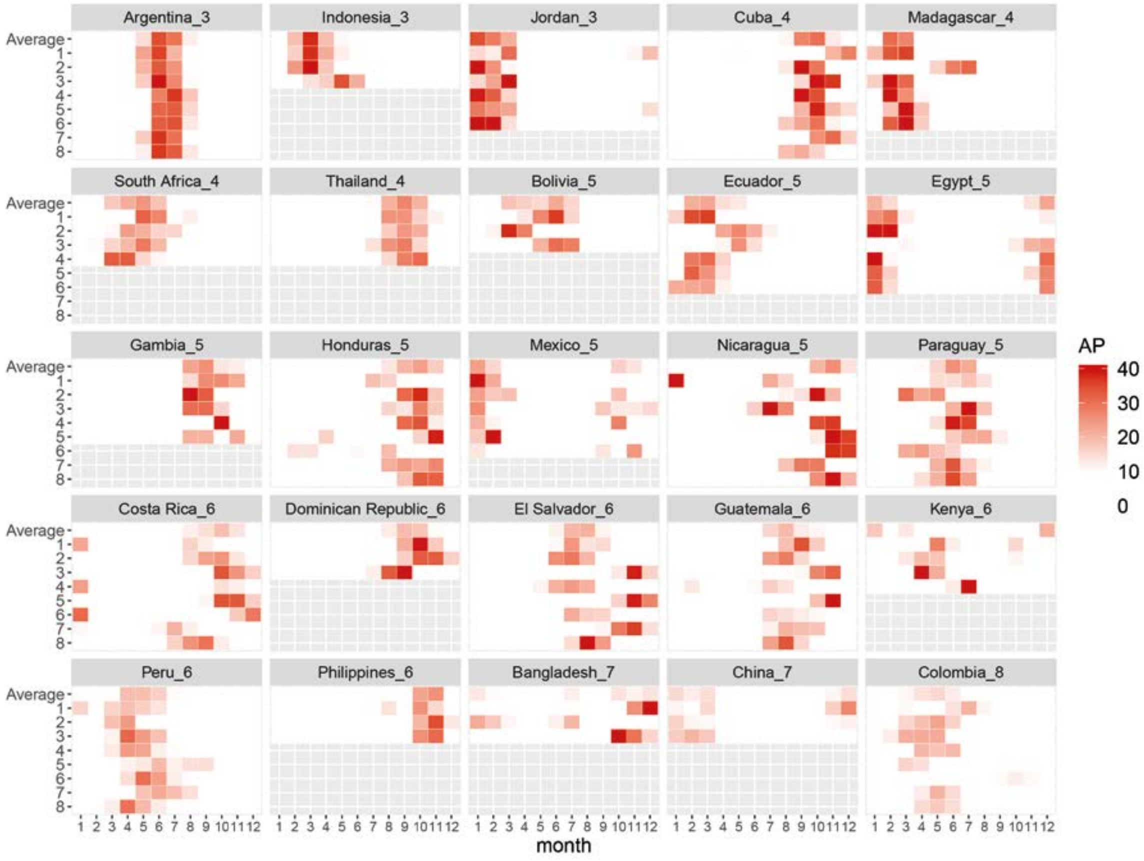

AP: annual percentage; LMICs are arranged by duration of average RSV seasonal epidemics. The months 1 to 12 represent January to December. Y-axis denotes the activity of each year with the annual average activity on the top.

Figure S5. Distribution of RSV-ALRI hospitalisation in infants <3m by birth month and by calendar month

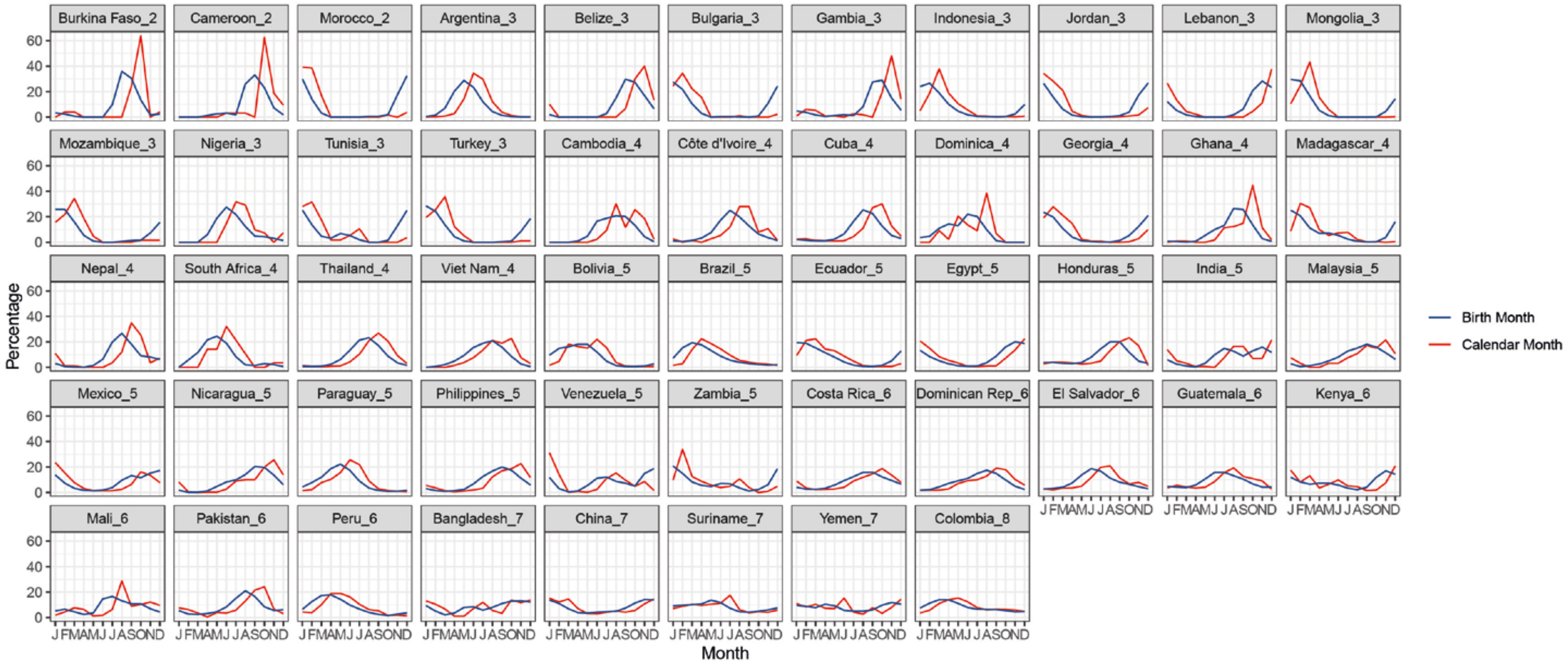

For each line, proportions of RSV-ALRI hospitalisation episodes add up to 100% across months. Countries are arranged by the duration of RSV epidemics (in months, shown next to country name).

Figure S6. Distribution of RSV-ALRI in infants <3m by birth month and by calendar month

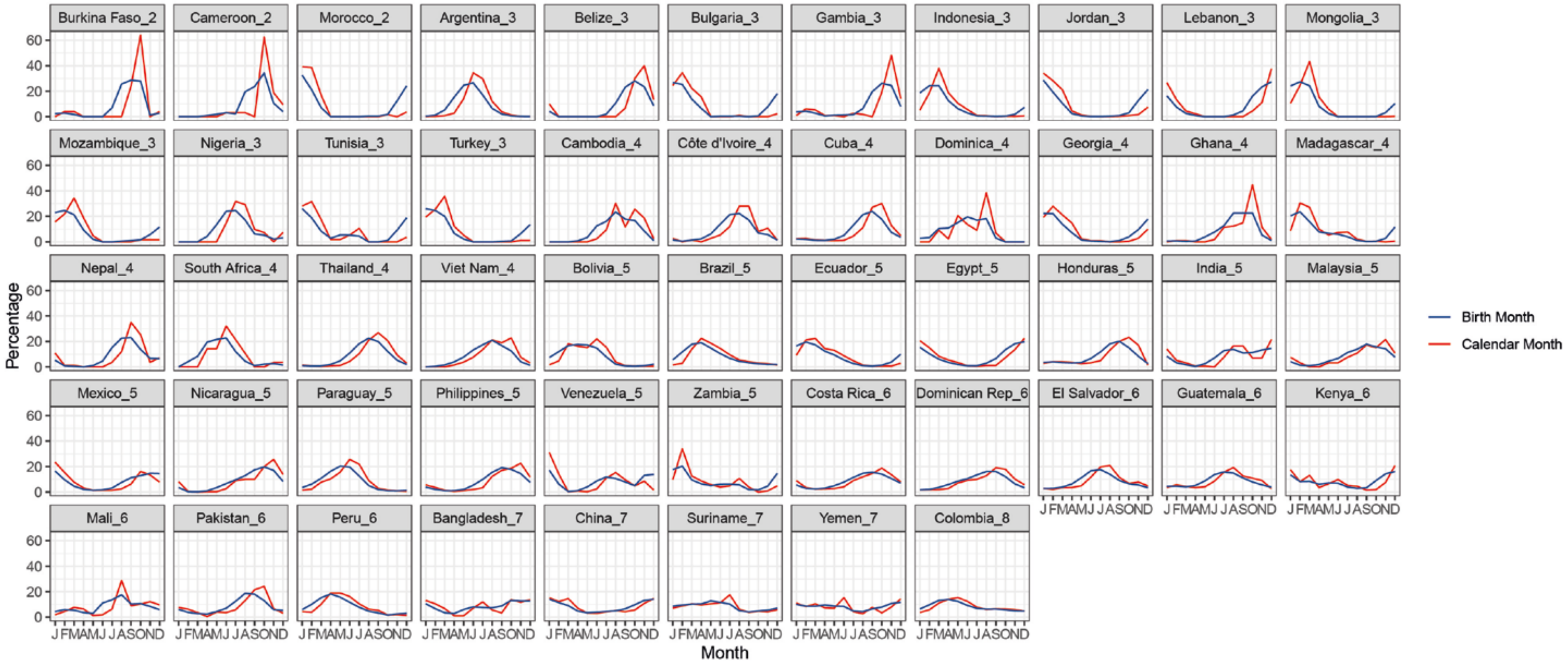

For each line, proportions of RSV-ALRI episodes add up to 100% across months. Countries are arranged by the duration of RSV epidemics (in months, shown next to country name).

Figure S7. Dosing schedules for seasonal mAb programmes

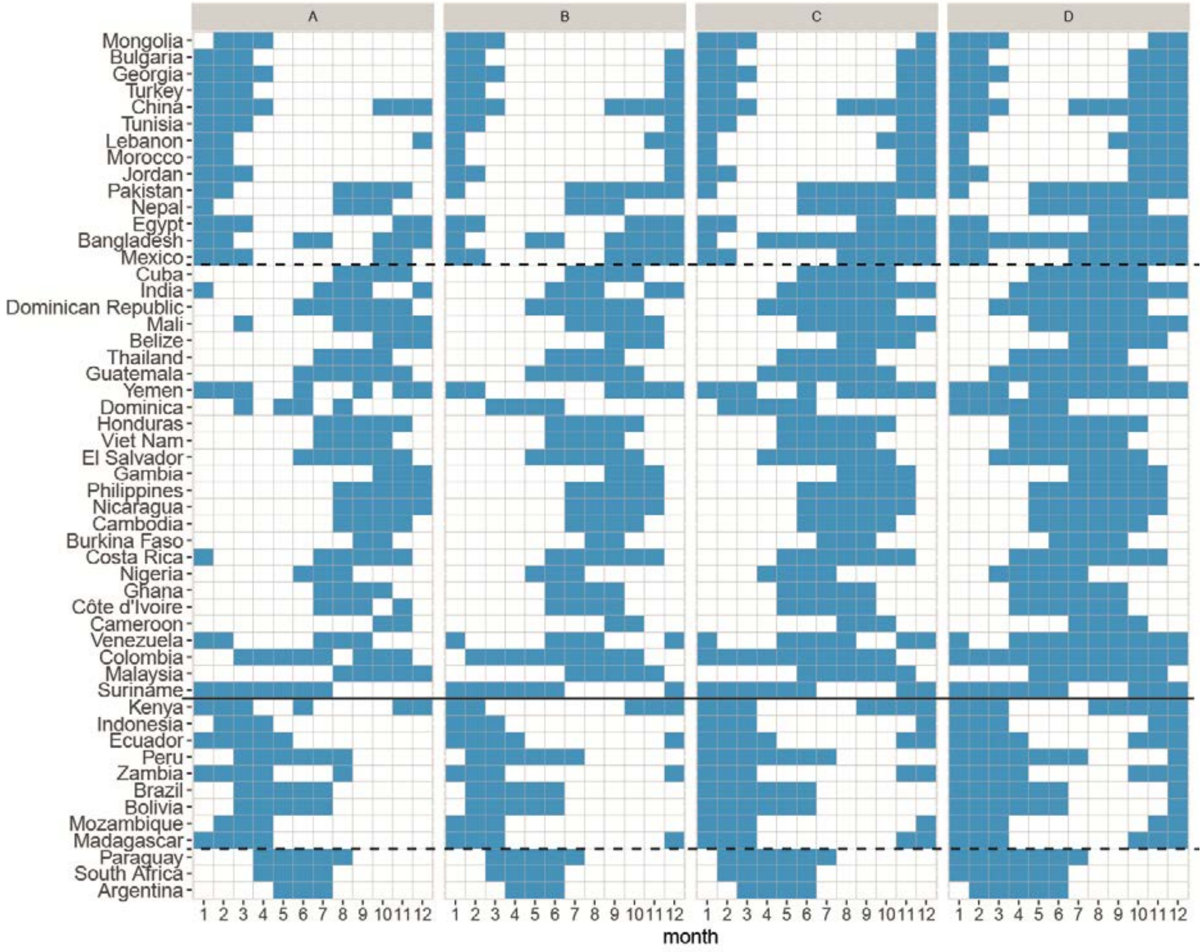

Countries arranged by latitude. The solid line denotes the equator and the dashed lines denote tropics of Cancer and Capricorn. Shaded areas denote mAb administration months.

Figure S8. Dosing schedules for seasonal maternal vaccine programmes

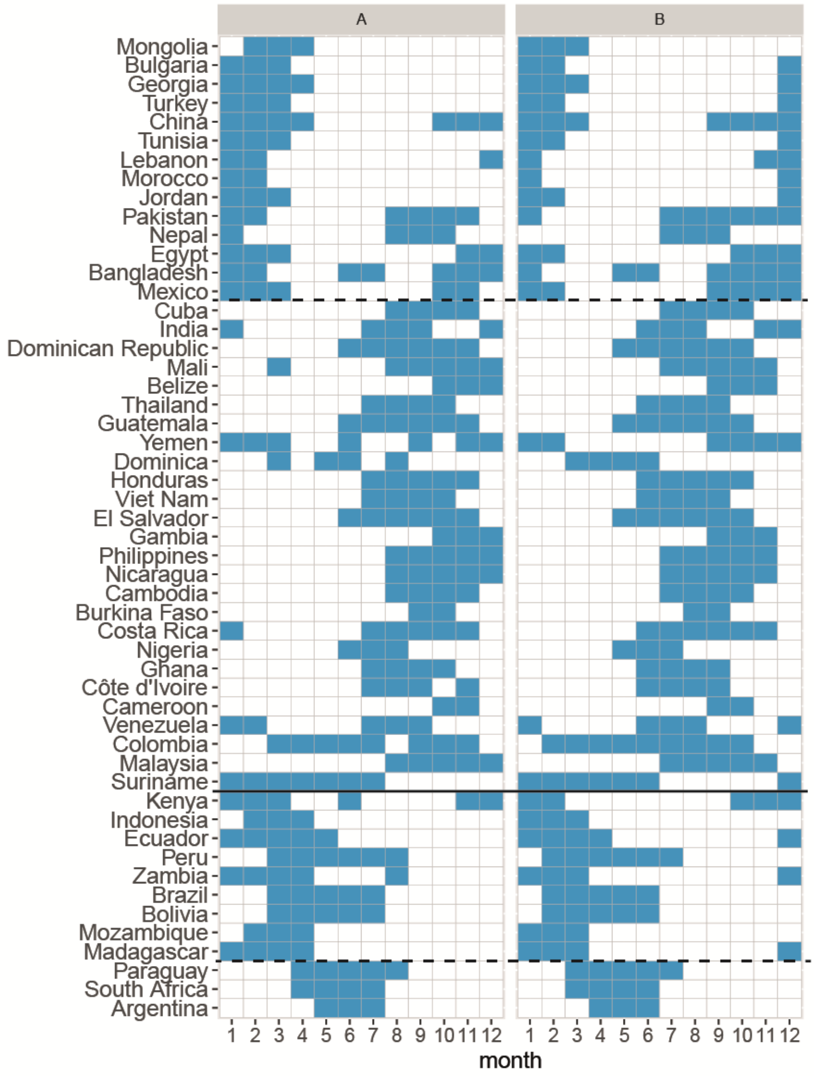

Countries arranged by latitude. The solid line denotes the equator and the dashed lines denote tropics of Cancer and Capricorn. Shaded areas denote birth months considered for the maternal vaccine programme.

Figure S9. Country-specific results of effectiveness and relative efficiency in averting RSV-ALRI for monoclonal antibodies

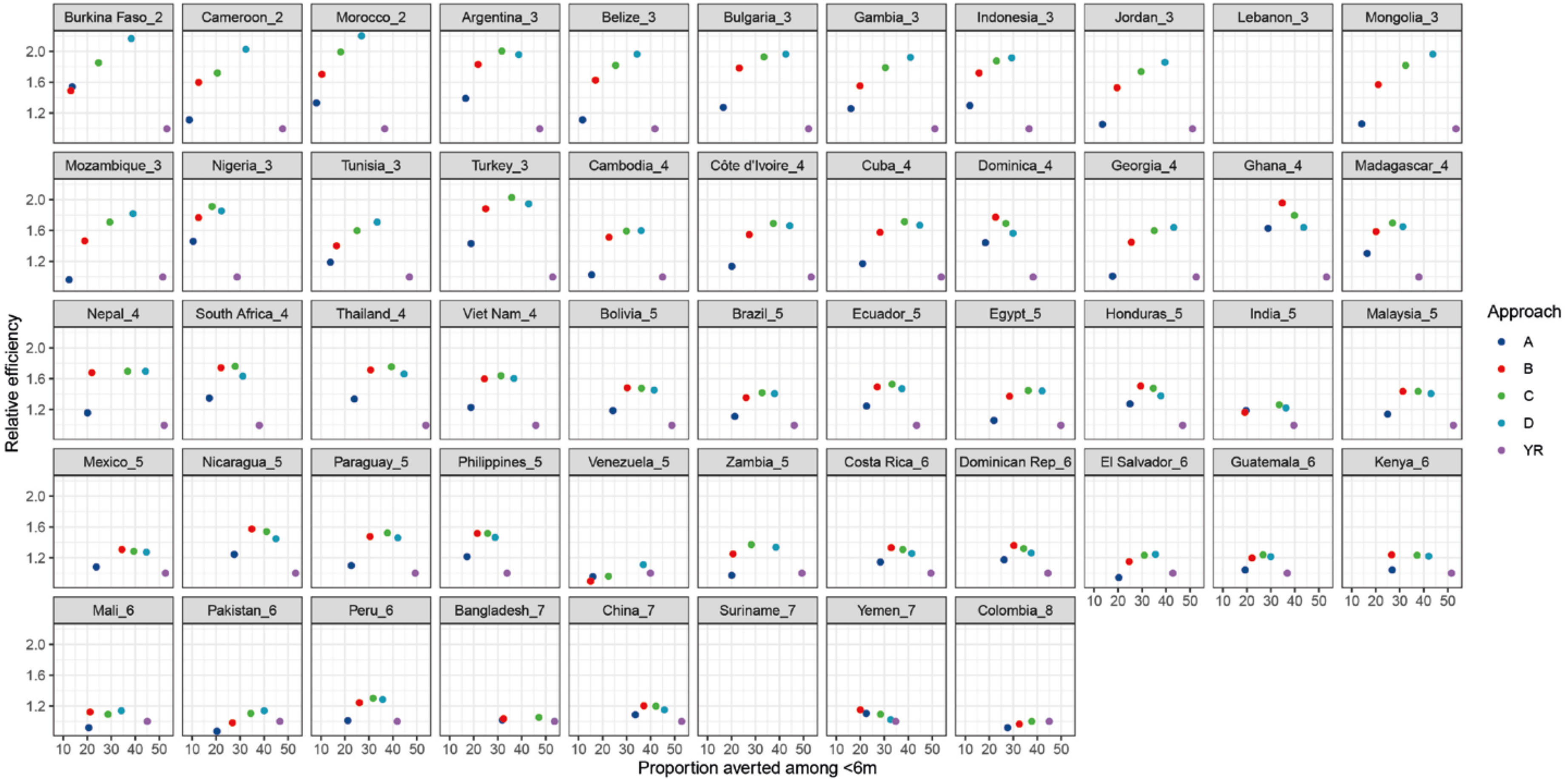

Number after each country indicates duration of RSV epidemics (in months). Effectiveness is defined by annual proportion averted among infants under six months of age; relative efficiency is defined by the ratio between per-dose effectiveness of a seasonal approach and that of the year-round approach. Approaches in the upper right quadrant would be considered those with optimal effectiveness and relative efficiency.

Figure S10. Country-specific results of effectiveness and relative efficiency in averting RSV-ALRI hospitalisation for maternal vaccine

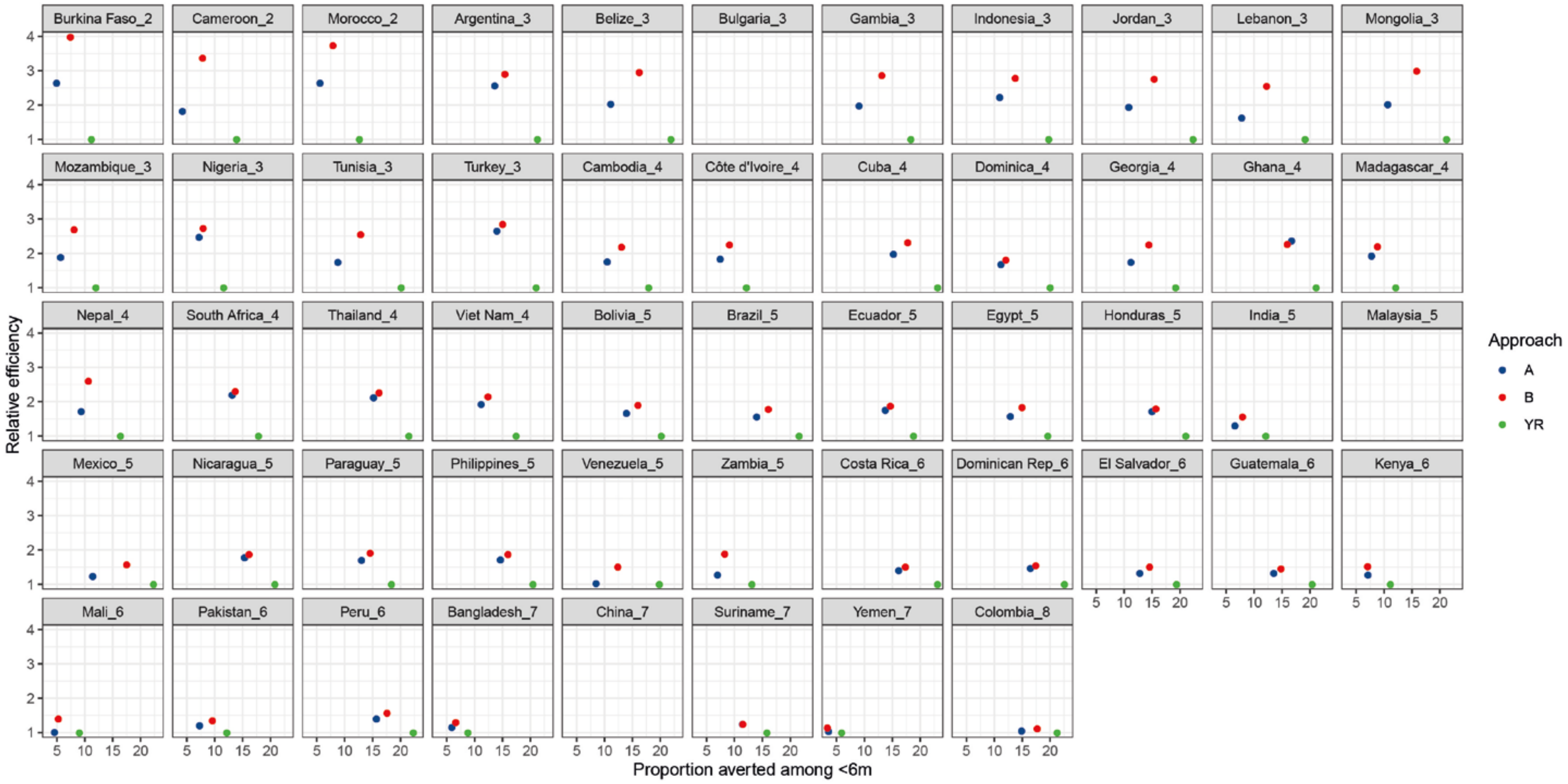

Number after each country indicates duration of RSV epidemics (in months). Effectiveness is defined by annual proportion averted among infants under six months of age; relative efficiency is defined by the ratio between per-dose effectiveness of a seasonal approach and that of the year-round approach. Approaches in the upper right quadrant would be considered those with optimal effectiveness and relative efficiency.

Figure S11. Country-specific results of effectiveness and relative efficiency in averting RSV-ALRI for maternal vaccine

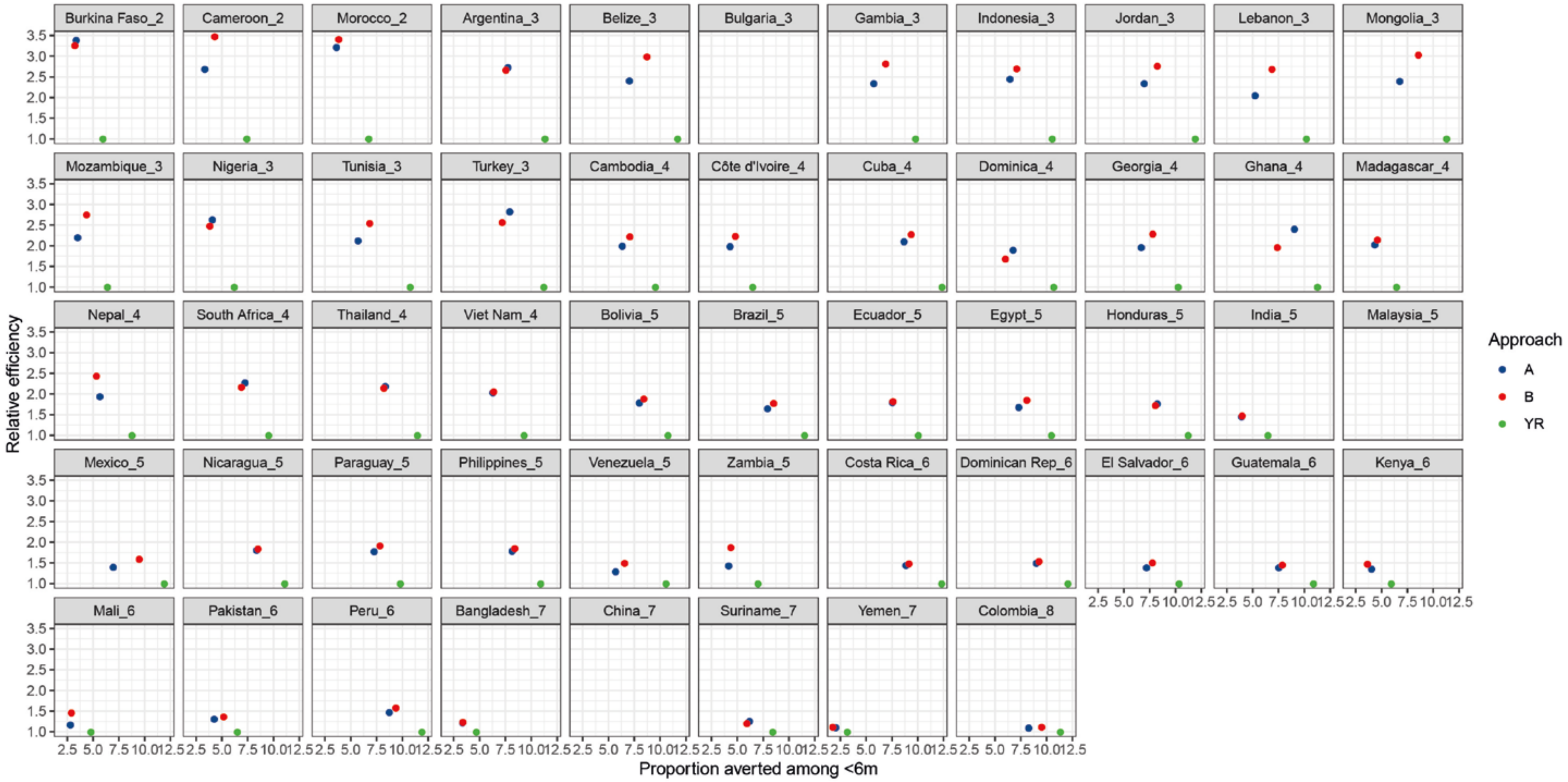

Number after each country indicates duration of RSV epidemics (in months). Effectiveness is defined by annual proportion averted among infants under six months of age; relative efficiency is defined by the ratio between per-dose effectiveness of a seasonal approach and that of the year-round approach. Approaches in the upper right quadrant would be considered those with optimal effectiveness and relative efficiency.

Figure S12. Year-to-year variations of effectiveness and relative efficiency in averting RSV-ALRI hospitalisation for monoclonal antibodies

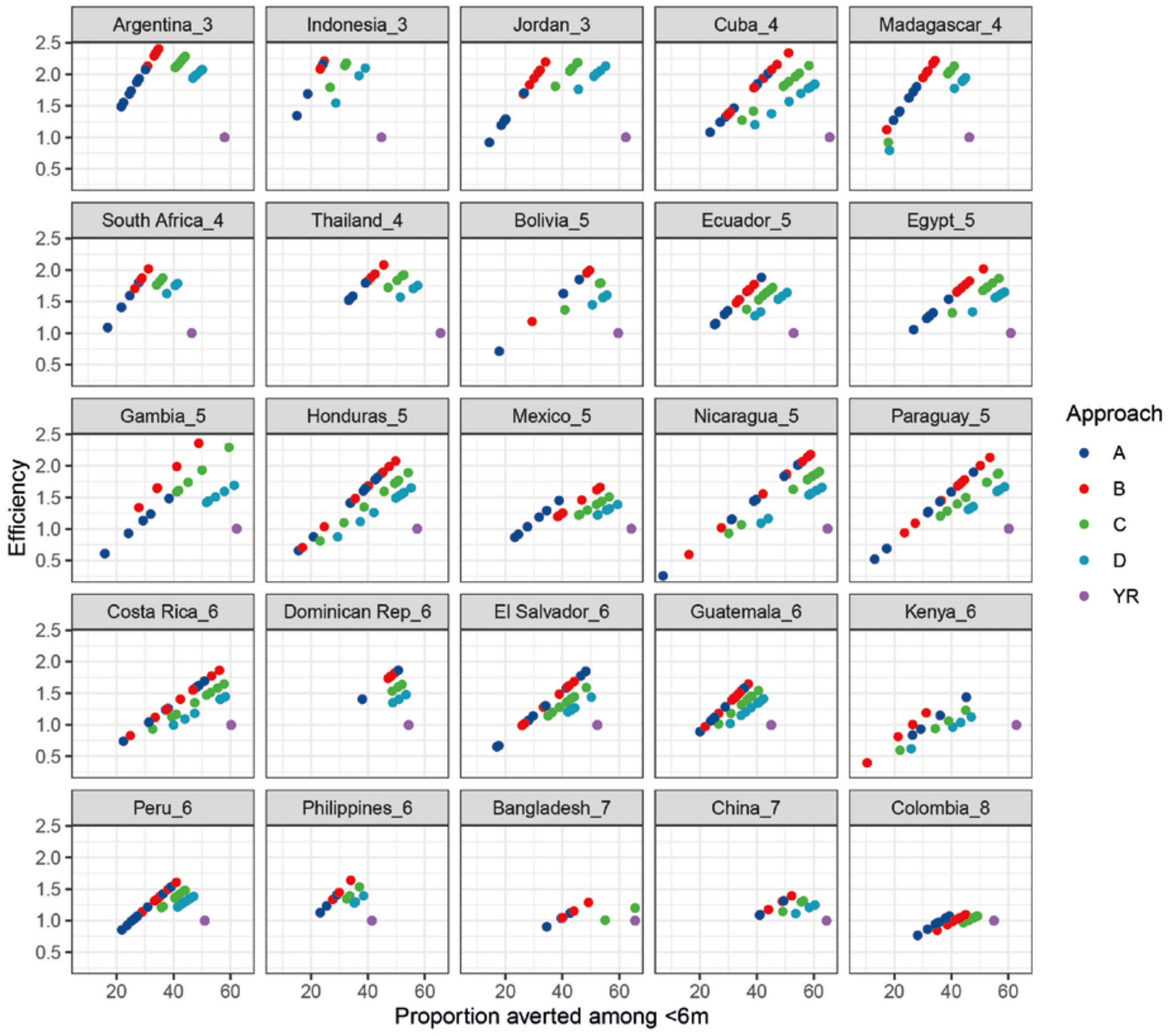

Countries are arranged by the duration of RSV epidemics (in months, shown next to country name). Each dot represents an approach in a single year. Effectiveness is defined by annual proportion averted among infants under six months of age; relative efficiency is defined by the ratio between per-dose effectiveness of a seasonal approach and that of the year-round approach. Approaches in the upper right quadrant would be considered those with optimal effectiveness and relative efficiency. Linear relationship between effectiveness and relative efficiency within each approach and country is due to the fact that relative efficiency is a function of effectiveness and number of dosing months; the latter is a constant for each approach and country. Degree of year-on-year variations can be reflected by the distance between dots of the same colour.

Figure S13. Year-to-year variations of effectiveness and relative efficiency in averting RSV-ALRI for monoclonal antibodies

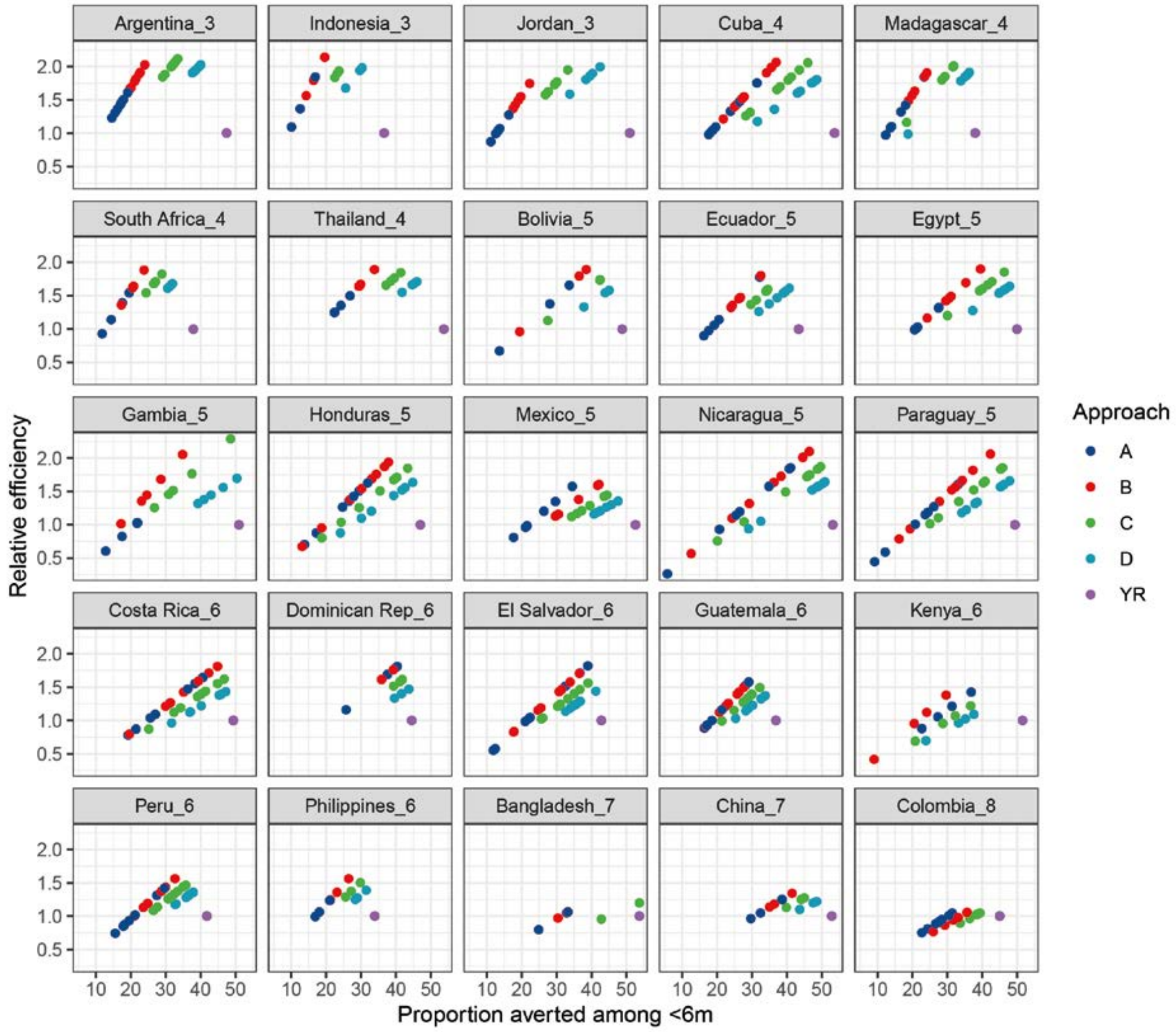

Countries are arranged by the duration of RSV epidemics (in months, shown next to country name). Each dot represents an approach in a single year. Effectiveness is defined by annual proportion averted among infants under six months of age; relative efficiency is defined by the ratio between per-dose effectiveness of a seasonal approach and that of the year-round approach. Approaches in the upper right quadrant would be considered those with optimal effectiveness and relative efficiency. Linear relationship between effectiveness and relative efficiency within each approach and country is due to the fact that relative efficiency is a function of effectiveness and number of dosing months; the latter is a constant for each approach and country. Degree of year-on-year variations can be reflected by the distance between dots of the same colour.

Figure S14. Year-to-year variations of effectiveness and relative efficiency in averting RSV-ALRI hospitalisation for maternal vaccine

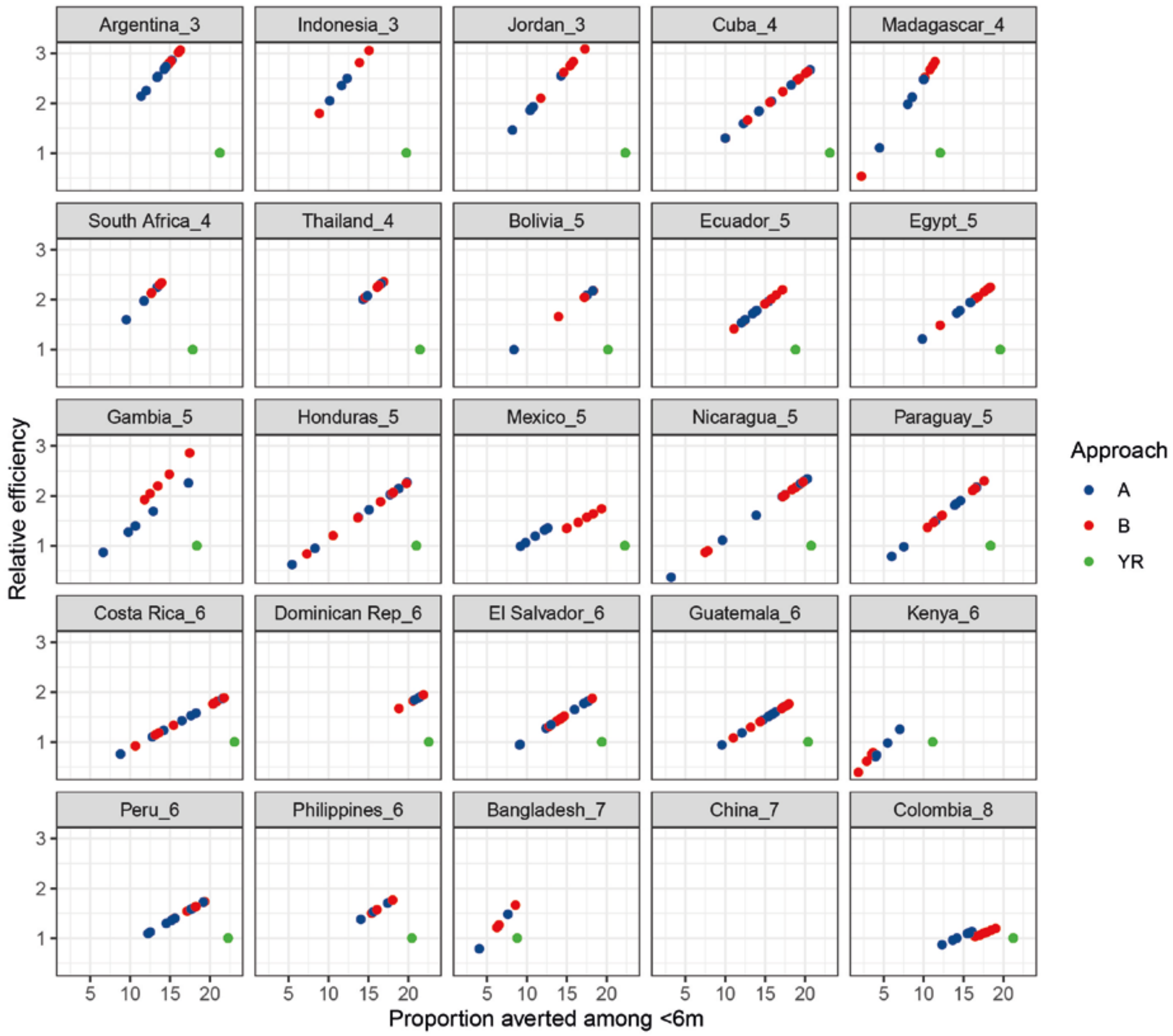

Countries are arranged by the duration of RSV epidemics (in months, shown next to country name). Each dot represents an approach in a single year. Effectiveness is defined by annual proportion averted among infants under six months of age; relative efficiency is defined by the ratio between per-dose effectiveness of a seasonal approach and that of the year-round approach. Approaches in the upper right quadrant would be considered those with optimal effectiveness and relative efficiency. Linear relationship between effectiveness and relative efficiency within each approach and country is due to the fact that relative efficiency is a function of effectiveness and number of dosing months; the latter is a constant for each approach and country. Degree of year-on-year variations can be reflected by the distance between dots of the same colour.

Figure S15. Year-to-year variations of effectiveness and relative efficiency in averting RSV-ALRI for maternal vaccine

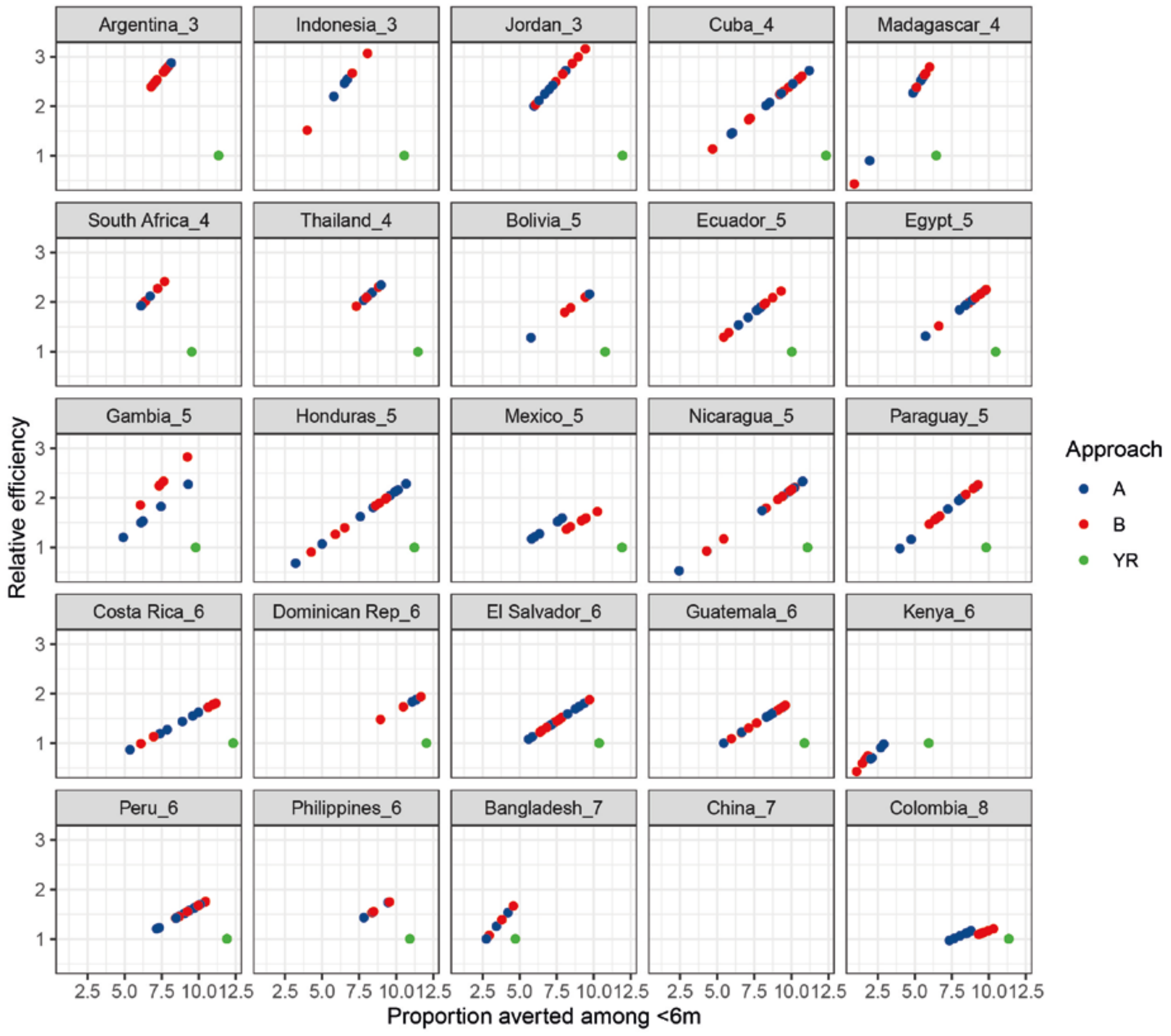

Countries are arranged by the duration of RSV epidemics (in months, shown next to country name). Each dot represents an approach in a single year. Effectiveness is defined by annual proportion averted among infants under six months of age; relative efficiency is defined by the ratio between per-dose effectiveness of a seasonal approach and that of the year-round approach. Approaches in the upper right quadrant would be considered those with optimal effectiveness and relative efficiency. Linear relationship between effectiveness and relative efficiency within each approach and country is due to the fact that relative efficiency is a function of effectiveness and number of dosing months; the latter is a constant for each approach and country. Degree of year-on-year variations can be reflected by the distance between dots of the same colour.

GATHER checklist

Checklist of information that should be included in new reports of global health estimates

Item #	Checklist item	Reported on page #
**Objectives and funding**		
**1**	Define the indicator(s), populations (including age, sex, and geographic entities), and time period(s) for which estimates were made.	4
**2**	List the funding sources for the work.	7
**Data Inputs**		
For all data inputs from multiple sources that are synthesized as part of the study:		
**3**	Describe how the data were identified and how the data were accessed.	4-5
**4**	Specify the inclusion and exclusion criteria. Identify all ad-hoc exclusions.	4-5
**5**	Provide information on all included data sources and their main characteristics. For each data source used, report reference information or contact name/institution, population represented, data collection method, year(s) of data collection, sex and age range, diagnostic criteria or measurement method, and sample size, as relevant.	4-5 & Appendix 3-6
**6**	Identify and describe any categories of input data that have potentially important biases (e.g., based on characteristics listed in item 5).	4-5
For data inputs that contribute to the analysis but were not synthesized as part of the study:		
**7**	Describe and give sources for any other data inputs.	N/A
For all data inputs:		
**8**	Provide all data inputs in a file format from which data can be efficiently extracted (e.g., a spreadsheet rather than a PDF), including all relevant meta-data listed in item 5. For any data inputs that cannot be shared because of ethical or legal reasons, such as third-party ownership, provide a contact name or the name of the institution that retains the right to the data.	Appendix 3-6
**Data analysis**		
**9**	Provide a conceptual overview of the data analysis method. A diagram may be helpful.	Figure S1
**10**	Provide a detailed description of all steps of the analysis, including mathematical formulae. This description should cover, as relevant, data cleaning, data pre-processing, data adjustments and weighting of data sources, and mathematical or statistical model(s).	6 & Appendix 8-9
**11**	Describe how candidate models were evaluated and how the final model(s) were selected.	N/A
**12**	Provide the results of an evaluation of model performance, if done, as well as the results of any relevant sensitivity analysis.	N/A
**13**	Describe methods for calculating uncertainty of the estimates. State which sources of uncertainty were, and were not, accounted for in the uncertainty analysis.	6-7
**14**	State how analytic or statistical source code used to generate estimates can be accessed.	7
**Results and Discussion**		
**15**	Provide published estimates in a file format from which data can be efficiently extracted.	Table 1 & Table S1-S
**16**	Report a quantitative measure of the uncertainty of the estimates (e.g. uncertainty intervals).	7-8

**17**	Interpret results in light of existing evidence. If updating a previous set of estimates, describe the reasons for changes in estimates.	9-10
**18**	Discuss limitations of the estimates. Include a discussion of any modelling assumptions or data limitations that affect interpretation of the estimates.	10-11

Reference

1. The World Bank. World Bank Country and Lending Groups. 2019. https://datahelpdesk.worldbank.org/knowledgebase/articles/906519-world-bank-country-and-lending-groups (accessed 22-January 2020).
2. Li Y, Reeves RM, Wang X, et al. Global patterns in monthly activity of influenza virus, respiratory syncytial virus, parainfluenza virus, and metapneumovirus: a systematic analysis. *The Lancet Global health* 2019; **7**(8): e1031-e45.
3. Appak O, Duman M, Belet N, Sayner AA. Viral respiratory infections diagnosed by multiplex polymerase chain reaction in pediatric patients. *Journal of medical virology* 2019; **91**(5): 731-7.
4. Chakhunashvili G, Wagner AL, Power LE, et al. Severe Acute Respiratory Infection (SARI) sentinel surveillance in the country of Georgia, 2015-2017. *PLoS ONE [Electronic Resource]* 2018; **13**(7): e0201497.
5. Chavez D, Gonzales-Armayo V, Mendoza E, et al. Estimation of influenza and respiratory syncytial virus hospitalizations using sentinel surveillance data-La Paz, Bolivia. 2012-2017. *Influenza and other respiratory viruses* 2019; **13**(5): 477-83.
6. Chittaganpitch M, Waicharoen S, Yingyong T, et al. Viral etiologies of influenza-like illness and severe acute respiratory infections in Thailand. *Influenza other respi* 2018; **12**(4): 482-9.
7. Kadjo HA, Adjogoua E, Dia N, et al. Detection of non-influenza viruses in acute respiratory infections in children under five-year-old in Cote D'Ivoire (January - December 2013). *African Journal of Infectious Diseases* 2018; **12**(2): 78-88.
8. Korsun N, Angelova S, Trifonova I, et al. Viral pathogens associated with acute lower respiratory tract infections in children younger than 5 years of age in Bulgaria. *Brazilian Journal of Microbiology* 2019; **50**(1): 117-25.
9. Li X, Li J, Meng L, et al. Viral etiologies and epidemiology of patients with acute respiratory infections based on sentinel hospitals in Gansu Province, Northwest China, 2011-2015. *Journal of medical virology* 2018; **90**(5): 828-35.
10. Li Y, Liang Y, Ling Y, Duan M, Pan L, Chen Z. The spectrum of viral pathogens in children with severe acute lower respiratory tract infection: a 3-year prospective study in the Pediatric Intensive Care Unit. *Journal of medical virology* 2019; **91**(9): 1633-42.
11. Liang X, Liu D, Chen D, et al. Gradual replacement of all previously circulating respiratory syncytial virus A strain with the novel ON1 genotype in Lanzhou from 2010 to 2017. *Medicine* 2019; **98**(19).
12. Obodai E, Odoom JK, Adiku T, et al. The significance of human respiratory syncytial virus (HRSV) in children from Ghana with acute lower respiratory tract infection: A molecular epidemiological analysis, 2006 and 2013-2014. *PLoS ONE [Electronic Resource]* 2018; **13**(9): e0203788.
13. Oladele DM, Oladele DP, Ibraheem RM, et al. Reappraisal of respiratory syncytial virus as an aetiology of severe acute lower respiratory tract infections in children younger than 5 years in Nigeria. *Transactions of the Royal Society of Tropical Medicine and Hygiene* 2019; **113**(8): 446-52.
14. Rabarison JH, Tempia S, Harimanana A, et al. Burden and epidemiology of influenza- and respiratory syncytial virus-associated severe acute respiratory illness hospitalization in Madagascar, 2011-2016. *Influenza other respi* 2019; **13**(2): 138-47.
15. Teck K, MacGuad R, Rostenberghe AHv, Hua G. Prevalence, risk factors and clinical characteristics of respiratory syncytial virus-associated lower respiratory tract infections in Kelantan, Malaysia. *Journal of medical virology* 2019; **91**(9): 1608-15.
16. Xu W, Guo L, Dong X, et al. Detection of Viruses and Mycoplasma pneumoniae in Hospitalized Patients with Severe Acute Respiratory Infection in Northern China, 2015-2016. *Japanese journal of infectious diseases* 2018; **71**(2): 134-9.
17. Yu J, Liu C, Xiao Y, et al. Respiratory syncytial virus seasonality, Beijing, China, 2007-2015. *Emerging infectious diseases* 2019; **25**(6): 1127-35.

18. Zeng Y, Yan H, Wang Q, Wang X. Detection and analysis of 9 pathogens in 6 984 cases with respiratory tract infection. *Journal of Tropical Medicine* 2018; **18**(11): 1440-3.
19. Shi T, McAllister DA, O'Brien KL, et al. Global, regional, and national disease burden estimates of acute lower respiratory infections due to respiratory syncytial virus in young children in 2015: a systematic review and modelling study. *The Lancet* 2017; **390**(10098): 946-58.
20. WHO. WHO-UNICEF estimates of BCG coverage. 2019. http://apps.who.int/immunization_monitoring/globalsummary/timeseries/tswucoveragebcg.html (accessed 02-February 2020).
21. WHO. Proportion of women aged 15-49 who received four or more antenatal care visits. 2019. http://apps.who.int/gho/data/view.xgswcah.3-data (accessed 02-February 2020).
22. Madhi SA, Polack FP, Piedra PA, et al. Respiratory Syncytial Virus Vaccination during Pregnancy and Effects in Infants. *New England Journal of Medicine* 2020; **383**(5): 426-39.
23. Griffin MP, Yuan Y, Takas T, et al. Single-Dose Nirsevimab for Prevention of RSV in Preterm Infants. *New England Journal of Medicine* 2020; **383**(5): 415-25.
